# Metabolomics Profiling of White Button, Crimini, Portabella, Lion’s Mane, Maitake, Oyster, and Shiitake Mushrooms Using Untargeted Metabolomics and Targeted Amino Acid Analysis

**DOI:** 10.3390/foods12162985

**Published:** 2023-08-08

**Authors:** Cassi N. Uffelman, Katrina A. Doenges, Michael L. Armstrong, Kevin Quinn, Richard M. Reisdorph, Minghua Tang, Nancy F. Krebs, Nichole A. Reisdorph, Wayne W. Campbell

**Affiliations:** 1 Department of Nutrition Science, Purdue University, West Lafayette, IN 47907, USA; cuffelma@purdue.edu; 2 Skaggs School of Pharmacy and Pharmaceutical Sciences, University of Colorado Anschutz Medical Campus, Aurora, CO 80045, USA; katrina.doenges@cuanschutz.edu (K.A.D.); michael.l.armstrong@cuanschutz.edu (M.L.A.); kevin.d.quinn@hotmail.com (K.Q.); richard.reisdorph@cuanschutz.edu (R.M.R.); nichole.reisdorph@cuanschutz.edu (N.A.R.); 3 School of Medicine, Department of Pediatrics, University of Colorado Anschutz Medical Campus, Aurora, CO 80045, USA; minghua.tang@cuanschutz.edu (M.T.); nancy.krebs@cuanschutz.edu (N.F.K.)

**Keywords:** *Agaricus bisporus*, *Pleurotus ostreatus*, *Hericium erinaceus*, *Grifola frondose*, *Lentinula edodes*, L-ergothioneine, fungi, food composition

## Abstract

Mushrooms contain multiple essential nutrients and health-promoting bioactive compounds, including the amino acid L-ergothioneine. Knowledge of the chemical composition of different mushroom varieties will aid research on their health-promoting properties. We compared the metabolomes of fresh raw white button, crimini, portabella, lion’s mane, maitake, oyster, and shiitake mushrooms using untargeted liquid chromatography mass spectrometry (LC/MS)-based metabolomics. We also quantified amino acid concentrations, including L-ergothioneine, a potential antioxidant which is not synthesized by plants or animals. Among the seven mushroom varieties, more than 10,000 compounds were detected. Principal Component Analysis indicated mushrooms of the same species, *Agaricus Bisporus* (white button, portabella, crimini), group similarly. The other varieties formed individual, distinct clusters. A total of 1344 (520 annotated) compounds were detected in all seven mushroom varieties. Each variety had tens-to-hundreds of unique-to-mushroom-variety compounds. These ranged from 29 for crimini to 854 for lion’s mane. All three *Agaricus bisporus* varieties had similar amino acid profiles (including detection of all nine essential amino acids), while other varieties had less methionine and tryptophan. Lion’s mane and oyster mushrooms had the highest concentrations of L-ergothioneine. The detection of hundreds of unique-to-mushroom-variety compounds emphasizes the differences in chemical composition of these varieties of edible fungi.

## 1. Introduction

Mushrooms have been consumed for thousands of years for nutritional and medicinal purposes. Mushrooms are low in energy and sodium, fat-free, cholesterol-free, and are considered an alternative source of moderate-to-high-quality protein [1,2]. They also contain fiber, B vitamins, selenium, potassium, glutathione, and L-ergothioneine [3]. Edible mushrooms are the primary dietary source of the amino acid, L-ergothioneine, which is not synthesized by higher plants or animals [4]. While the physiological role of L-ergothioneine has not been fully elucidated, it is proposed as an adaptive antioxidant which may protect against the tissue damage implicated in several chronic diseases [5,6,7]. L-ergothioneine is also proposed as a “longevity vitamin” that may promote healthy aging, though further research is needed [8].

In addition to essential nutrients, mushrooms have several bioactive compounds, including polysaccharides, lectins, terpenoids, sterols, and alkaloids, among others, which may positively impact health [9]. The cell walls of mushrooms contain polysaccharides, including β-glucans and chitin, which positively affect health, e.g., through the modulation of the immune system and protection of the cardiovascular system through improvements in glucose and lipid metabolism [10]. Effects on the cardiovascular system are also attributable to lovastatin and polyphenols, known for their lipid-lowering and antioxidant properties, respectively [11,12]. Fungal lectins, which have several biological roles, including cellular signaling, have attracted attention for their immunomodulatory, antiproliferative, and antitumor activities [13]. While terpenoids are a large class of compounds found throughout nature, their therapeutic uses span multiple physiological processes, including anti-inflammatory, antioxidant, and anticancer processes [14,15,16]. Alkaloids produced in mushrooms have biological activities including antioxidant, antibacterial, anti-inflammatory, and neuroprotective properties, among others described in a 2022 review [17]. Thus, mushrooms and their bioactive extracts are considered functional foods [18].

While untargeted metabolomics has been performed on several mushroom varieties [19,20,21,22,23,24], this is, to our knowledge, the first to compare the metabolomes of seven commonly consumed mushroom varieties. Therefore, the purpose of this research is to document the metabolomic profiles of seven different mushroom varieties using an untargeted metabolomics approach employing liquid chromatography mass spectrometry (LC/MS). Additionally, given that the literature supports the role of amino acids in the potential health benefits of mushrooms, we aim to quantify amino acid concentrations, including glutathione and L-ergothioneine, using a targeted approach. Knowing the chemical composition of mushrooms will enhance knowledge regarding the potential mechanisms of action responsible for human health impacts. Additionally, the findings from this work will help inform ongoing randomized clinical trials of mushroom consumption (NCT04257201 and NCT04259229).

## 2. Materials and Methods

### 2.1. Untargeted Metabolomics

#### 2.1.1. Chemicals, Standards, and Reagents

All solvents used for sample preparation and LC/MS analysis were of high-performance liquid chromatography (HPLC) or LC/MS grade. These included water from Honeywell Burdick & Jackson (Muskegon, MI, USA), methyl tert-butyl ether (MTBE) from VWR (Radnor, PA, USA), formic acid from ThermoFisher Scientific (Waltham, MA, USA), acetonitrile and methanol from Fisher Scientific (Hampton, NH, USA), 2-Propanol from Millipore Sigma (Burlington, MA, USA), and InfinityLab Deactivator Additive from Agilent Technologies (Santa Clara, CA, USA). Authentic standards for sample preparation were from Avanti Polar Lipids Inc. (Alabaster, AL, USA), Cambridge Isotope Laboratories (Tewksbury, MA, USA), Sigma-Aldrich (St. Louis, MO, USA) and CDN Isotopes (Pointe-Claire, Quebec, Canada). Amino acid standards were from Sigma and Pickering Laboratories (Mountain View, CA, USA).

#### 2.1.2. Mushroom Procurement

Seven mushroom varieties were sourced from three farms in the United States of America, using two farms per variety. Farm A provided all seven mushroom varieties, including *Agaricus bisporus* (white button, crimini, portabella), *Hericium erinaceus* (lion’s mane), *Pleurotus ostreatus* (oyster), *Grifola frondose* (maitake), and *Lentinula edodes* (shiitake); Farm B sourced mushrooms of the species *Agaricus bisporus* (white button, crimini, portabella); and Farm C sourced specialty mushrooms, *Hericium erinaceus* (lion’s mane), *Pleurotus ostreatus* (oyster), *Grifola frondose* (maitake), and *Lentinula edodes* (shiitake) (Table 1). All mushrooms were harvested within a week of each other in November 2020, and were immediately shipped fresh to the analytical laboratory at University of Colorado Anschutz Medical Campus. Upon receipt (in Aurora), mushrooms were stored at 4 °C and processed within 48 h.

#### 2.1.3. Mushroom Sample Processing and Homogenization

Prior to processing, mushrooms were rinsed for 10 s and patted dry with a Kimwipe (Kimberly-Clark Professional, Corinth, MS, USA) to remove substrate residues. White button, crimini, and shiitake mushroom samples had approximately 3 mm of their stems removed with a clean knife, and the portabella mushroom samples were split in half and only one half of each was processed. All mushroom varieties from each farm were prepared in triplicate and diced individually in a clean food processor for approximately 10 s, or until very small chunks were present. The food processor was thoroughly cleaned between samples by rinsing with tap water, deionized water, and finally methanol to prevent cross-contamination. A total of 50–100 mg of each diced mushroom sample was weighed into pre-chilled Qiagen 2 mL Tissue Lyser tubes with steel beads (Hilden, Germany) and stored on dry ice. Ice-cold methanol (−20 °C) was added to each sample at a rate of 100 µL methanol to 10 mg mushroom. Samples were homogenized with a Qiagen TissueLyser LT for 2 min at 50 Hz, followed by centrifugation at 0 °C for 15 min at 18,000× *g* (Beckman Coulter, Brea, CA, USA) to pellet proteins and particulates. A total of 100 µL of the supernatants were transferred to 1.5 mL microfuge tubes and stored at −80 °C until liquid-liquid extraction. The remaining supernatants were transferred to 1.5 mL microfuge tubes and stored at −80 °C for targeted amino acid/L-ergothioneine analysis. Two process blanks were prepared alongside mushroom samples by blending deionized (DI) water or methanol for 10 s in the food processor. A 1 mL aliquot of each was stored at –80 °C for sample preparation.

#### 2.1.4. Mushroom Sample Preparation

A modified MTBE liquid-liquid extraction protocol was used to separate the hydrophobic and hydrophilic fractions of each mushroom sample for untargeted metabolomics, as described previously [25,26,27,28]. Briefly, samples were spiked with 10 µL of both Avanti’s SPLASH Lipidomix and an in-house hydrophilic spike mix (L-acetylcarnitine-d_3_:HCl, ^13^C_5_-adenosine (ribose), adenosine triphosphate-d_4_ (ammonium salt, ribose), L-alanine-d_3_, L-aspartic acid-d_3_, carnitine-d_3_:HCl, creatinine-d_3_, U-^13^C_6_-D-glucose, U-^13^C_6_-D-glucose-6-phosphate (disodium salt, hydrate), L-lysine-d_4_:HCl, methyl-d_3_-malonic acid, ^13^C_6_-niacinamide, succinic acid-d_6_ and L-valine-d_8_ at 10 µg/mL and L-lactate-d_3_ (sodium salt) at 2 µg/mL, in 50:50 methanol:water). A total of 400 µL ice-cold methanol was added to the mushroom homogenate aliquots to aid in protein precipitation. After vortexing to mix and centrifugation (15 min at 18,000× *g* and 0 °C), supernatants were transferred to glass culture tubes and dried under Nitrogen at 35 °C. MTBE and water were added to the glass tubes, vortexed, and centrifuged (10 min, room temperature, 1000× *g*). The top hydrophobic (MTBE) layer was transferred to a clean culture tube. A second addition of MTBE was added to the first culture tube, vortexed and centrifuged as before, and the top layer was combined in the second culture tube. Both the hydrophobic and hydrophilic tubes were dried under nitrogen at 35 °C. The hydrophobic fraction was reconstituted immediately in methanol, transferred to autosampler vials (Cornerstone Scientific, Leland, NC, USA), and stored at −80 °C until analysis. The dried hydrophilic fraction underwent a second protein precipitation with water and ice-cold methanol, and the supernatant was dried by speed vac at 45 °C. Samples were reconstituted in 5% acetonitrile in water and stored at −80 °C until analysis. Aliquots from a subset of prepped samples, representing mushrooms from each variety and farm, were pooled together to make the instrument QCs on the day of the instrumental analysis, for both the hydrophobic and hydrophilic fractions. Spiked and un-spiked methanol preparation blanks, as well as spiked plasma samples (Innovative Research, Novi, MI, USA), were prepared alongside mushroom samples in each daily preparation batch. Two spiked process blanks were also prepared alongside samples in the final batch. Plasma samples were used for preparation QC purposes.

#### 2.1.5. Hydrophobic Liquid Chromatography Mass Spectrometry (LC/MS)

The hydrophobic fraction was analyzed using an Agilent 6545 liquid chromatography-quadrupole time-of-flight mass spectrometer (LC-QTOF-MS) (Agilent Technologies, Santa Clara, CA, USA). The hydrophobic fractions of all mushroom samples were analyzed using reverse-phase chromatography with an Agilent Zorbax Rapid Resolution HD (RRHD) SB-C18, 1.8 µL (2.1 mm × 100 mm) analytical column. The injection volume was 5 µL with a flow rate of 0.7 mL/min. The mobile phase A included water with 0.1% formic acid and the mobile phase B included 60:36:4 2-propanol:acetonitrile:water with 0.1% formic acid. The mobile phase gradient was as follows: 0–0.5 min 70% B, 0.5–7.42 min 70–100% B, 7.42–10.4 min 100% B, 10.4–10.5 min 100–70% B, 10.5–15.1 70% B. The autosampler tray temperature was set to 4 °C and the column temperature was set to 60 °C [27].

The MS conditions for the hydrophobic mushroom samples were as previously described, including the LC-QTOF-MS run in positive ionization mode, scan rate of 2 spectra/s, mass range *m*/*z* 75–1700, drying gas temperature 300 °C and flow rate of 12.0 L/min, nebulizer pressure 35 psi, sheath gas temperature 275 °C, sheath gas flow 12 L/min, skimmer 65 V, capillary voltage 3500 V, fragmentor 100 V, and reference masses 121.050873 and 922.009798 (Reference mix, Agilent Technologies) [27].

#### 2.1.6. Hydrophilic Liquid Chromatography Mass Spectrometry (LC/MS)

The hydrophilic fraction was analyzed using an Agilent 6560 Ion Mobility liquid chromatography-quadrupole time-of-flight mass spectrometry (LC-IM-QTOF-MS) (Agilent Technologies, Santa Clara, CA, USA), run in QTOF mode only (i.e., no ion mobility). The aqueous fractions of all mushroom samples were analyzed with a Zorbax SB-AQ C18, 5 µm (2.1 mm × 100 mm) analytical column. The injection volume was 5 µL with a flow rate of 0.25 mL/min. Mobile phase A included 0.1% formic acid in water with 0.1% InfinityLab Deactivator Additive, and mobile phase B included 0.1% formic acid in 90% aqueous Acetonitrile with 0.1% InfinityLab Deactivator Additive. The mobile phase gradient was as follows for positive mode: 0–3.0 min 1.8% B, 3–10 min 1.8–54% B, 10–15 min 54–90% B, 15–20 min 90% B, 20–20.1 min 90–1.8% B, 20.1–25 min 1.8% B. The autosampler tray temperature was set to 4 °C and the column temperature was set to 30 °C.

The MS conditions for the hydrophilic mushroom samples were as follows: LC-IM-QTOF-MS was run in positive ionization mode, scan rate 2.0 spectra/second, mass range *m/z* 50–1700, gas temperature 300 °C, gas flow 12.0 L/min, nebulizer 35 psi, skimmer 65 V, capillary voltage 3500 V, fragmentor 100 V, reference masses 121.050873 and 922.009798 (Reference mix, Agilent Technologies).

#### 2.1.7. Data Processing

Untargeted raw data were extracted using a recursive workflow with Mass Hunter Profinder version B.10 Service Pack 1 (Profinder, Agilent Technologies) and compound peak heights were imported into Mass Profiler Professional version 15.1 (MPP, Agilent Technologies) for analysis [27,28]. Prior to analysis, compounds found in preparation and process blanks were removed from the dataset. Data from hydrophobic and hydrophilic fractions were extracted separately using Batch Molecular Feature Extraction (BMFE) followed by Batch Targeted Feature Extraction (BTFE) in Profinder. Hydrophobic data were extracted as previously reported [27] with the following modifications: noise peak height filter ≥ 10,000 counts, alignment tolerance for RT was 0% + 0.15 min with a mass of 10 ppm + 2 mDa, and absolute height peak filter ≥ 20,000 counts with a score of ≥80 for BMFE, and peak filter height ≥ 13,000 counts with a score ≥ 50 for BTFE. Hydrophilic data were extracted similarly to the hydrophobic fraction with the following modifications: RT extraction range of 0–20.0 min, noise peak height filter ≥ 2000 counts and absolute peak height filter ≥10,000 counts with a score of ≥80 for BMFE, and peak filter height ≥ 6000 counts with a score ≥ 50 for BTFE. These thresholds are based on manual review of the data to distinguish noise from signal and to determine peak widths [29]. After importing into MPP for analysis, compounds were filtered to be present in at least one mushroom sample to retain all compound information.

#### 2.1.8. Compound Annotation

Agilent MassHunter ID Browser version 10.0 (ID Browser) was used to annotate compounds using in-house and commercial databases. The databases can be broken down into three broad categories and were searched in this order: (1) an in-house accurate mass and retention time database created from Mass Spectrometry Metabolite Library standards (IROA Technologies, Ann Arbor, MI, USA), using both the hydrophobic and hydrophilic instrument methods; (2) in-house food databases comprising data from FooDB, Phenol Explorer and plant compounds from the Human Metabolome Database (HMDB); and (3) in-house biological databases comprising data from HMDB, Lipid Maps, Kyoto Encyclopedia of Genes and Genomes (KEGG), and METLIN. Annotation parameters were as reported previously [27]. In the event that two masses with different retention times were assigned the same compound name, the label “Esi + time” appears in the name of the compound eluting at a later time. Manual interpretation of data has previously determined that these are unlikely to be the same compound. However, because no other annotations were available, in order to ensure that distinct compounds were compared using statistics, the software-generated annotations were retained. Annotated names correspond to a Metabolomics Standards Initiative (MSI) level three and are considered putative [30]. Compound annotations are used for the purpose of comparing compounds across mushroom subspecies and are putative; compound names are not used to imply structural or chemical similarities between compounds or mushroom subspecies.

#### 2.1.9. Data Visualization

Raw data (i.e., before statistics) for compounds that were present in at least one mushroom sample were visualized using principal component analysis (PCA) and hierarchical clustering (HC) in MPP, as previously described [27,28].

#### 2.1.10. Statistical Analysis

Statistical analysis of compounds detected using untargeted metabolomics was performed in MPP. One-way ANOVAs were conducted on mushroom samples (n = 3 replicates per variety per farm) with Tukey’s post hoc and Benjamini–Hochberg false discovery rate (FDR) (*p* < 0.05) to identify compounds that are significantly different between the mushroom varieties.

Statistical analysis of amino acids (n = 3 replicates per variety per farm) was performed in R Studio using R v.3.5.1. Amino acids that were below the limit of quantitation were given a value of 0. Four ANOVA models were fitted (Mushroom Variety; Farm; Mushroom Variety + Farm; and Mushroom Variety + Mushroom Variety: Farm). The ANOVA model with the lowest Akaike Information Criterion (AIC) for each compound was used in the subsequent analysis. Tukey’s Honestly Significant Difference (HSD) was used to determine the difference in means by mushroom variety and identify compounds that are significantly different between varieties (*p* < 0.05). The least squares means, according to the best fit ANOVA model, was used to obtain the means and standard error (SE) for each compound by variety.

#### 2.1.11. Compound Curation and Identification of Potential Mushroom-Specific Compounds

Compound curation included manually researching the annotated compounds to describe their compound classification (category/superclass, main class, subclass) and to characterize them as a food/mushroom-specific compound, described in detail below.

Using an approach to identify “food-specific compounds” developed by Reisdorph et al. (2020) [28], our team researched each annotated compound detected in the mushroom sample replicates and categorized them as “Previously determined to be in that food (i.e., that mushroom variety)”, “Probably/Possibly in that food (identified in mushrooms generally)”, “Found in some/any other food”, “Natural product (not known to be found in mushrooms or foods)”, “Other (putatively annotated as exogenous, non-natural products)”, or “Cannot determine (i.e., not enough information)”. Each compound was re-searched using HMDB (https://hmdb.ca/, first accessed on 9 May 2022), FooDB (https://foodb.ca/, first accessed on 9 May 2022), KEGG (https://www.genome.jp/kegg/, first accessed on 9 May 2022), and Lipid Maps (https://www.lipidmaps.org/, first accessed on 9 May 2022) for information regarding the previous detection of the compound in that mushroom variety. A summary of findings from the listed databases, Google, and PubMed (https://pubmed.ncbi.nlm.nih.gov/, first accessed on 9 May 2022) was recorded and used to categorize each compound. A list of potential mushroom-specific compounds was then generated by looking at compounds found in all seven varieties, unique to white button, and unique to oyster mushrooms categorized as “Previously determined to be in that food (i.e., that mushroom variety)”, or “Probably/Possibly in that food (identified in mushrooms generally)”. To be considered a potential mushroom-specific compound, the compound could not be described as detected in or associated with other foods.

Compounds could also be categorized as a “Natural product that is not known to be found in mushrooms or foods” (e.g., a bacterial product) or as “Exogenous, non-natural products” (e.g., a pollutant) or as “Cannot determine, not enough information”. Because exogenous compound annotations are less likely to be accurate, database search results were manually reviewed. For example, a compound with the molecular formula C18H26O2 was matched to mass rodinyl phenylacetate and apo-13-zeaxanthione. The former is a synthesized floral fragrance agent that is insoluble in water and therefore unlikely to be detected in the aqueous fraction, whereas apo-13-zeaxanthione is a terpenoid that has been found in several plants and foods. Therefore, the final putative compound annotation was designated as apo-13-zeaxanthione.

### 2.2. Amino Acid Analysis

#### 2.2.1. Sample Preparation

Preparation of the mushroom samples for amino acid analysis included adding 10 µL of mushroom homogenate, 10 µL of 0.1N HCL, 10 µL of U^13^C-Yeast internal standard (Cambridge Isotope Laboratories, Tewksbury, MA, USA), 10 µL of PBS buffer, and 120 µL of methanol to a 1.5 mL microfuge tube. Next, the samples were vortexed for 5 s and centrifuged at 18,000× *g* for 10 min at 4 °C. The supernatant was transferred to a new microfuge tube and then dried in a vacuum centrifuge (Labconco, Kansas City, MO, USA) for 45 min at 45 °C. Samples were reconstituted immediately with 100 µL of 0.05N HCL, vortexed for 10 s, and then centrifuged at 18,000× *g* for 5 min. Finally, the supernatant was removed and placed into an amber autosampler vial with a 250 µL glass insert (Agilent Technologies).

#### 2.2.2. Hydrophilic Interaction Liquid Chromatography—Triple Quadrupole Mass Spectrometry (HILIC-QQQ-MS)

Extracted mushroom samples were analyzed as previously described [31,32] with an Agilent Technologies Poroshell 120 HILIC-Z 2.1 × 100 mm, 2.7 µm analytical column. The injection volume was 1 µL with a flow rate of 0.8 mL/min. Mobile phase A was 20 mM ammonium acetate pH = 3.2 in water and mobile phase B was 20 mM ammonium acetate pH = 3.2 in 90:10 acetonitrile:water. The mobile phase gradient was as follows: 100% B to 70% B over 10.00 min, hold at 30% B from 10 min to 11 min, then re-equilibrate at 100% B for 5 min. The MS conditions were as follows: Agilent 6490 triple quadrupole (QQQ-MS) with JetStream source in positive mode, gas temperature 290 °C, gas flow 11 L/min, nebulizer 35 psi, sheath gas temperature 390 °C, sheath gas flow 11 L/min, capillary voltage 3500 V, fragmentor 380 V, ion funnel high pressure RF 150, low pressure RF 60. Raw data (i.e., before statistics) for amino acids were acquired in MRM mode using experimentally optimized conditions obtained using flow injection analysis of authentic standards. Quantitation was performed using a routine internal standard method. All target analytes and internal standards were monitored for quantitation by extracting ion chromatograms for each respective MRM transition and calculating the peak area using MassHunter Quantitative Analysis Software (Agilent Technologies). Calibration curves for each analyte were calculated using linear regression or quadratic fit with a 1/x weighting. Results were normalized to the wet weight of the sample prior to homogenization.

Concentrations of amino acids in mushroom homogenate was calculated using the following equation:

$$\mathrm{Concentration}\;\mathrm{in}\;\mathrm{mg}/100\;g=\frac{(Xs)(Vt)(D)*100}{\left(Vi)(Ws\right)}$$

where:

Xs = measured concentration in mg/mL;
Vt = total volume of concentrated extract (in mL);
D = dilution factor if sample was extracted before analysis;

If no dilution D = 1;

Vi = volume of extract injected (in µL);
Ws = weight of sample extracted in g.

## 3. Results

### 3.1. Untargeted Metabolomics Analysis Detects Thousands of Compounds in Mushrooms

**Figure 1:**
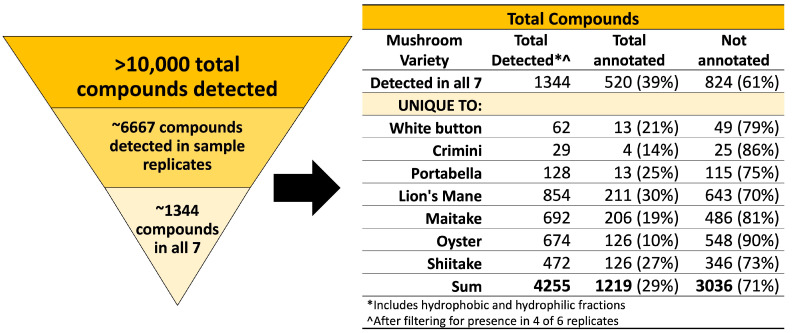
Summary of compounds detected in the seven mushroom varieties. Over 10,000 compounds were detected in at least 1 of the 42 mushroom samples from seven mushroom varieties. Results were filtered for presence of the compound in at least 4 of 6 sample replicates (n = 6667). Next, compounds of interest were those detected in all seven mushroom varieties or unique-to-mushroom-variety (n = 4255).

A total of 42 samples from seven mushroom varieties were analyzed using an untargeted metabolomics approach. As described in the methods (Section 2.1.8), compound annotations are used to putatively compare compounds across mushroom subspecies. Compound names are not used to imply structural or chemical similarities between compounds or mushroom subspecies. We detected 10,144 different compounds (1806 in the hydrophobic fraction and 8338 in the hydrophilic fraction) in at least one of the 42 samples. Next, we filtered the results to identify compounds detected in all seven mushroom varieties and compounds that are unique to each mushroom variety. We set the filter such that the compound must be detected in four of six sample replicates to reduce the number of potential extraction artifacts and to improve the confidence of the compound being endogenous to the mushroom(s). This filtering process resulted in 6667 total compounds detected in ≥4 sample replicates, of which 1344 (520 annotated) were detected in all seven mushroom varieties, and 2911 (699 annotated) were unique to a specific mushroom variety, as summarized in Figure 1. Mushroom compounds detected in sample replicates (all seven mushrooms or unique-to-mushroom-variety) are available in Supplemental File S1.

**Figure 2:**
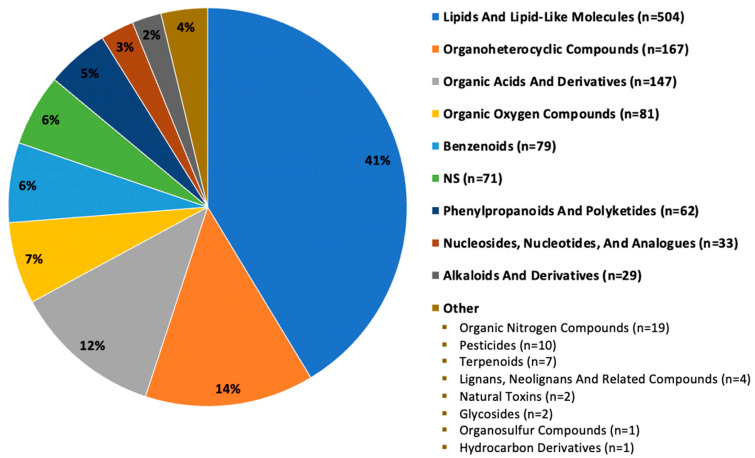
Superclass categorization of 1219 compounds detected in seven mushroom varieties. Note: n = 1219 compounds (hydrophobic and hydrophilic fractions) are those detected in all seven mushroom varieties or unique-to-mushroom-variety (required presence in 4 of 6 sample replicates).

Of the 1219 annotated compounds (hydrophobic and hydrophilic fractions) detected in all seven mushrooms or unique-to-mushroom-variety, 41% (n = 504) were classified as lipid and lipid-like molecules. Other major categories included: organoheterocyclic compounds; organic acids and derivatives; organic oxygen compounds; benzenoids; phenylpropanoids and polyketides; nucleosides, nucleotides, and analogues; and alkaloids and derivatives, as depicted in Figure 2. About 6% (n = 71) of compounds did not have a compound classification available, designated by NS for not specified. Categories reported fewer than 20 times were grouped in the “other” label for simplicity. Supplemental Figure S1 depicts select bioactive compounds, including various polyphenols, terpenoids, and phytosterols detected in our samples.

### 3.2. Mushroom Samples Group Based on Species as Depicted by Principal Component Analysis (PCA) and Hierarchical Clustering (HC)

**Figure 3:**
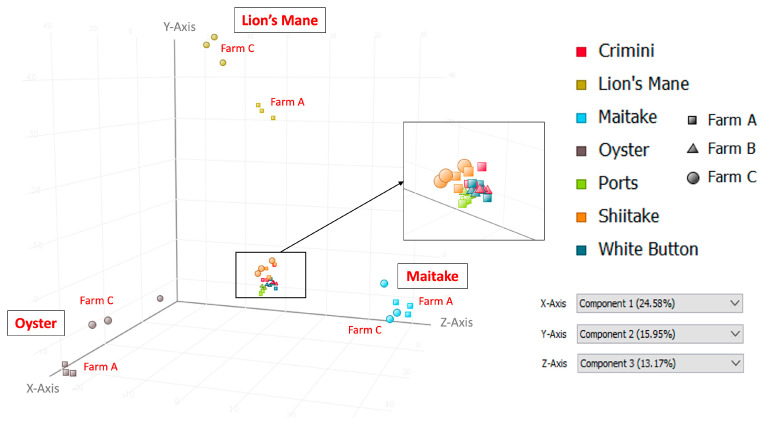
Principal Component Analysis (PCA) using data from the hydrophobic fraction of seven mushroom varieties. Component 1, which explains 24.58% of the variation, is shown on the *x*-axis; component 2, which explains 15.95% of the variation, is shown on the *y*-axis; and component 3, which explains 13.17% of the variation, is shown on the *z*-axis.

**Figure 4:**
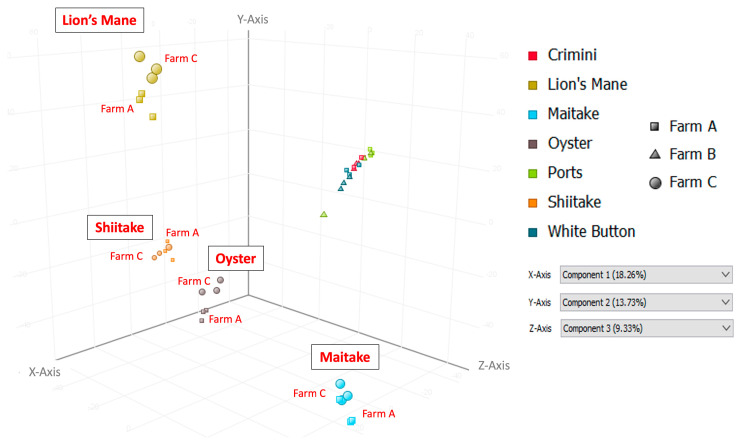
Principal Component Analysis (PCA) using data from the hydrophilic fraction of seven mushroom varieties. Component 1, which explains 18.26% of the variation, is shown on the *x*-axis; component 2, which explains 13.73% of the variation, is shown on the *y*-axis; and component 3, which explains 9.33% of the variation, is shown on the *z*-axis.

Principal component analysis of the 10,144 compounds detected in at least 1 of the 42 mushroom samples from seven mushroom varieties (in both hydrophobic and hydrophilic fractions) indicates that mushrooms cluster by species, regardless of the farm (Figure 3 and Figure 4). Mushrooms of the same species, *A. bisporus*, including white button, crimini, and portabella, group together.

**Figure 5:**
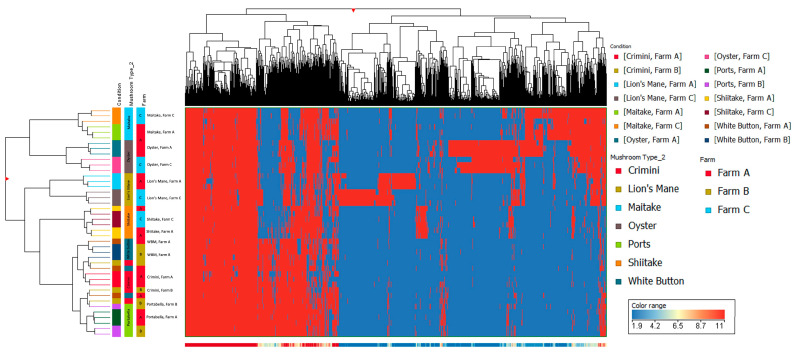
Hierarchical clustering using data from the hydrophobic fraction of seven mushroom varieties. The *x*-axis corresponds to individual compounds detected in the hydrophobic fraction of the mushroom samples, which are grouped by variety and farm on the *y*-axis. The blue areas indicate less relative abundance of a compound, while the red areas indicate higher relative abundance of a compound compared to the other 1806 compounds. The vertical distance between compounds roughly estimates their similarity (e.g., a greater vertical difference indicates less similarity).

**Figure 6:**
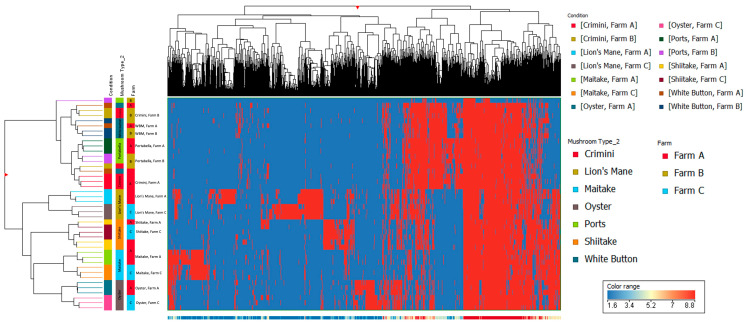
Hierarchical clustering using data from the hydrophilic fraction of seven mushroom varieties. The *x*-axis corresponds to individual compounds detected in the hydrophilic fraction of the mushroom samples, which are grouped by variety and farm on the *y*-axis. The blue areas indicate less relative abundance of a compound, while the red areas indicate higher relative abundance of a compound compared to the other 8338 compounds. The vertical distance between compounds roughly estimates their similarity (e.g., a greater vertical difference indicates less similarity).

Other mushroom varieties were distinctly clustered, suggesting that mushroom variety is the main driver of differences in mushroom condition and not farm. These findings are also supported by the hierarchical clustering of the seven different mushroom varieties in both hydrophobic and hydrophilic fractions, such that there were regions of distinct variation between the different mushroom species (i.e., *A. bisporus* compared to the other four mushroom varieties, lion’s mane, oyster, maitake, and shiitake) (Figure 5 and Figure 6). Within *A. bisporus*, the three mushroom varieties were clustered, supporting the similarities in their chemical composition illustrated in the PCA plots.

### 3.3. Statistical Analysis—One-Way ANOVA

We completed one-way ANOVAs with Tukey’s post hoc and Benjamini–Hochberg FDR (*p* < 0.05) to identify compounds that are significantly different between varieties. Among the compounds detected in sample replicates of any mushroom variety, 5690 compounds (hydrophobic and hydrophilic fractions) passed the ANOVA thresholds (Table 2). We observed striking differences in the number of significantly different compounds between specialty mushrooms (i.e., lion’s mane, maitake, oyster, shiitake) and varieties of the species, *A. bisporus* (white button, crimini, portabella). For example, there were 2327 compounds that differed between lion’s mane and crimini mushrooms. In contrast, 255 and 285 compounds were significantly different between crimini and portabella or white button mushrooms, respectively. One-way ANOVAs were also run on compounds (hydrophobic and hydrophilic fractions) detected in sample replicates of all seven mushroom varieties. There were 549 total compounds that passed ANOVA thresholds (Table 3). Hundreds of compounds were significantly different between specialty mushrooms and *A. bisporus* varieties (i.e., 212 differed between lion’s mane and crimini). Conversely, less than 50 compounds passed the ANOVA thresholds for comparisons between white button, crimini, and portabella mushroom varieties. Compounds detected in all seven mushroom varieties that passed the ANOVA thresholds can be found in Supplemental File S3.

### 3.4. Several Compounds Are Unique-to-Mushroom-Variety

We detected 2911 (699 annotated) unique-to-mushroom-variety compounds (hydrophobic and hydrophilic fractions; Figure 1, Supplemental File S1). Lion’s mane, maitake, oyster, and shiitake mushroom varieties each had more than 400 unique-to-mushroom-variety compounds. In contrast, unique-to-mushroom-variety compounds detected in portabella, white button, and crimini were 128, 62, and 29, respectively.

### 3.5. Untargeted Metabolomics Analysis Reveals Potential Mushroom-Specific Compounds

Through extensive manual interpretation, data were used to determine the likelihood of the annotated compound being unique to mushrooms. As described in the Section 2, compounds of interest were those that were categorized as “Previously determined to be in that food”, or “Probably/possibly in that food” (Table 4). Further criteria for consideration included the compound of interest not being reported as detected in or associated with other foods or in any of the test foods provided to participants in our ongoing acute feeding study (NCT04257201).

Briefly, untargeted metabolomics analysis revealed eight potential mushroom-specific compounds among white button, oyster, and all seven mushroom varieties (Table 5). One compound, (3beta,5alpha,9alpha,22E,24R)-5,9-epidioxy-3-hydroxyergosta-7,22-dien-6-one, and an isomer of this compound were detected in both oyster and white button mushrooms, respectively. For simplicity, the compound and its isomer are considered a single potential mushroom-specific compound. One other compound, ergosterol peroxide, was detected in white button mushrooms, while three other compounds were detected in oyster mushrooms, including 2-acetoxy-3-geranylgeranyl-1,4-dihydroxybenzene, methyl (Z,Z)-10-hydroxy-2,8-decadiene-4,6-diynoate, and polyporusterone E Esi + 0.83. Three potential mushroom-specific compounds were detected in all seven mushroom varieties including cerebroside B, N-(2R-Hydroxyhexadecanoyl)-2S-amino-9-methyl-4E,8E-octadecadiene-1,3R-diol Esi + 4.5509977, and (3beta,22E,24R)-Ergosta-4,6,8(14),22-tetraen-3-ol Esi + 15.40999.

### 3.6. Amino Acid Profiles Vary among Different Mushroom Varieties

**Figure 7:**
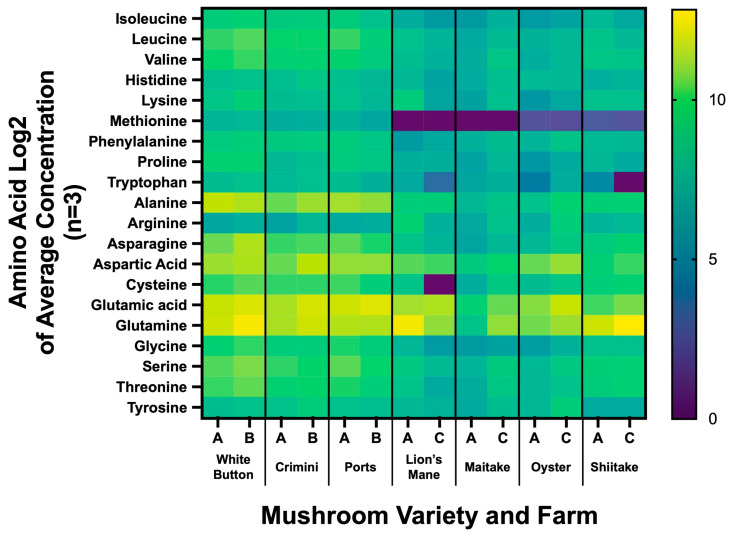
Amino acid profiling of seven mushroom varieties sourced from two different farms. Heatmap results are displayed as the Log2 of the average concentration of the 3 sample replicates from each farm. The *x*-axis indicates the mushroom variety and whether it was sourced from farm A, B, or C (e.g., White button was sourced from farm A and B).

We found the amino acid profiles to vary greatly among the different mushroom varieties (Figure 7, Supplemental File S4). Consistent with the results from the untargeted metabolomics analysis, the amino acid profiles were similar among mushrooms of the species *A. bisporus* (white button, portabella, crimini). The other four mushroom varieties (lion’s mane, maitake, oyster, and shiitake) had lower concentrations of methionine and tryptophan. Glutathione concentrations ranged from 6.20 ± 8.89 mg/100 g (mean ± SD) in lion’s mane to 162.24 ± 36.23 mg/100 g in maitake. The concentrations of the thirty different amino acids by mushroom variety and farm are available in Supplemental File S2.

### 3.7. L-Ergothioneine Concentration Varies among Different Mushroom Varieties

**Figure 8:**
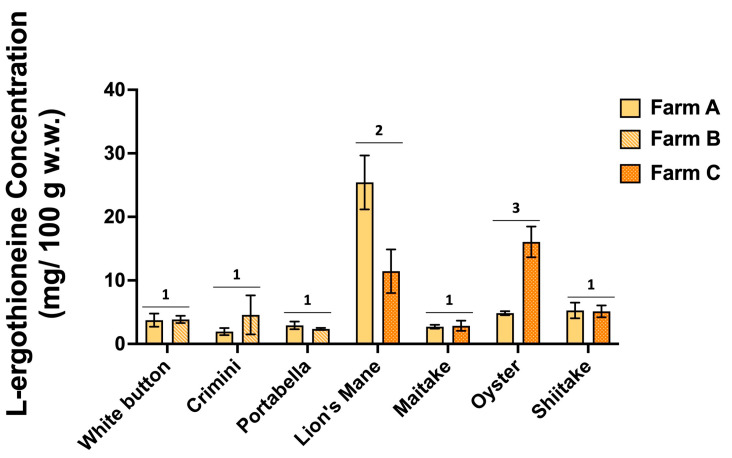
L-ergothioneine concentrations (mg/100 g; normalized to wet weight) in the seven mushroom varieties sourced from two different farms. Different numbers denote significance (*p <* 0.05).

The concentration of L-ergothioneine varies widely among the different mushroom varieties (Figure 8, Supplemental File S4). Results of ANOVA indicate significantly higher concentrations of L-ergothioneine in lion’s mane and oyster mushrooms compared to the remaining five mushroom varieties, which had concentrations ranging from 1.94 ± 0.55 to 5.26 ± 1.23 mg/100 g (mean ± SD). There was also variability in the concentration of L-ergothioneine between mushroom varieties of the same farm. For example, L-ergothioneine was significantly different between the farms producing lion’s mane and oyster mushrooms.

## 4. Discussion

Using an untargeted metabolomics approach, we assessed the chemical composition of seven different mushroom varieties, each sourced from two different farms. We detected over 10,000 different compounds among the mushroom samples, of which 1344 (520 annotated) compounds were detected in all seven mushroom varieties. Tens to hundreds of unique-to-mushroom-variety compounds were revealed, highlighting that there are genuine differences in their chemical composition (Figure 1). Using a targeted approach, we also found the amino acid profiles to vary among the different mushroom varieties. Consistent with previous reports, we also confirmed lion’s mane and oyster mushrooms have the highest L-ergothioneine concentrations (Figure 7 and Figure 8, and Supplemental File S4) [35,36].

To our knowledge, this is the most comprehensive assessment of the chemical composition of these seven common mushroom varieties conducted to date. A strength of this study is that we are effectively creating a “library” of compounds detected in mushrooms that will guide future mechanistic and targeted metabolomics work. Given the novelty of this work, a majority of the compounds detected among the seven mushroom varieties have not previously been detected in or related to mushrooms (Table 4). Further explained, <2% (28 of 1219) of the annotated compounds in sample replicates were categorized as “previously determined to be in that food” while <13% (160 of 1219) were categorized as “probably/possibly in that food”, using existing metabolomics databases and/or the published literature (described in Supplemental File S1) at the time of the study, highlighting the need for continued work on this topic and underscoring the complicated nature of the relation between consuming whole foods, such as mushrooms, and metabolic health.

We identified eight potential mushroom-specific compounds in white button, oyster, or all seven mushroom varieties (Table 5). Limited evidence has confirmed the detection of three of these compounds in various mushroom varieties. Ergosterol peroxide has been identified in several edible mushrooms including lion’s mane (16.0 ± 0.78 mg/100 g dry weight) [34] and oyster, and is described as a secondary metabolite with anti-inflammatory, anticancer, and antiviral properties, among others, in animal and cell models [33,37]. Polyporusterone E, detected in the fruiting body of *Polyporus umbellatus*, is reported to exhibit cytotoxic activity on leukemia 1210 cell proliferation [38]. Cerebroside B, detected in *Meripilus giganteus*, has possible antioxidant properties (oxygen radical absorbance capacity [ORAC] 1.69 ± 0.20 mmol TE/g) [39] and has demonstrated inhibitory effects on the proliferation of human breast cancer in MCF-7 cells [40]. Cerebroside B concentrations have been described for several edible mushroom species including king oyster (0.95 mg/g dry weight), pearl oyster (1.06 mg/g dry weight), and white button (0.59 mg/g dry weight) [41]. While limited evidence suggests compounds in mushrooms may have potential nutraceutical properties, these findings are based on compounds isolated from mushrooms and not on the use of whole, fresh, dietary mushrooms. Thus, further research is needed to assess the concentrations of these bioactive compounds in whole mushrooms, their bioavailability upon consumption in humans, and their physiological impact with acute and chronic consumption.

Bioactive compounds, including polyphenols, terpenoids, and phytosterols, have been identified in mushrooms previously and were also detected in our samples [42]. Polyphenols, further classified into phenolic acids, flavonoids, stilbenes, and lignans, are important antioxidants that may protect against the development of several chronic diseases, including diabetes, cancer, and cardiovascular diseases [43]. As depicted in Supplemental Figure S1, a majority of the polyphenolic compounds among our mushroom samples were varieties of phenolic acids (derivatives of benzoic acid or cinnamic acid) and flavonoids. We detected 18 compounds categorized as benzoic acids and derivatives, 17 cinnamic acids and derivatives, and 15 hydroxycinnamic acids and derivatives. Over 30 compounds were categorized as flavonoids, of which three were detected in all seven mushroom varieties. Oyster, shiitake, and lion’s mane mushrooms had five, eight, and thirteen unique-to-mushroom-variety flavonoids, respectively. More than 80 terpenoid compounds, including monoterpenoids, sesquiterpenoids, diterpenoids, and triterpenoids, were detected among our mushroom samples. The therapeutic and medicinal uses of isolated terpenoids in vivo are related to their anti-inflammatory, anti-metastatic, anti-angiogenesis, and apoptosis-inducing properties [14,15]. Note that terpenes are volatile and may have degraded during processing; in addition, this class of compounds is usually analyzed using gas chromatography whereas liquid chromatography was used in the current study. Phytosterols are plant sterols that have cholesterol-lowering properties and thus may have protective effects against the development of cardiovascular diseases [44]. Common phytosterols found in the diet are sitosterol, campesterol, and stigmasterol. We found derivatives of sitosterol and campesterol as 22:3-Glc-Sitosterol in all seven mushroom varieties and 22:2-Glc-Campesterol in oyster mushrooms.

Notable compounds unique to lion’s mane mushrooms include hericene (A, B, and C), hericenone (B, C, D, and E), hericerin, and herierin IV (Supplemental File S1). Hericenes and hericenones have neuroprotective effects against tunicamycin- and thapsigargin-induced endoplasmic reticulum stress-dependent Neuro2a (murine neuroblastoma) cell death [45,46]. Hericerin, a related compound, has neurotrophic properties through the increased production of nerve growth factor in C6 glioma cells [47]. Hericerin has also demonstrated anticancer properties by reducing the cell proliferation of HL-60 human acute promyelocytic leukemia cells, suggesting the potential use of this compound for cancer treatment [48]. Results from a recent study indicate that herierin IV, among other compounds from lion’s mane mushroom, exerts antidepressant effects in a mouse model of depression (induced by chronic restraint stress) by promoting neurogenesis and reducing neuroinflammation [49]. These findings highlight that the lion’s mane mushroom contains compounds which may have important implications for brain health, though research in humans is needed.

Mushrooms have previously been described as a novel, alternative source of moderate-to-high-quality protein [1,2,50]. Our work demonstrates that amino acid concentrations are vastly different among the different species (Figure 7, Supplemental File S4). *A. bisporus* (white button, crimini, and portabella) mushroom varieties were found to have all nine essential amino acids. While methionine and tryptophan concentrations measured the lowest among the nine essential amino acids in *A. bisporus* mushrooms, there was a complete absence of methionine detected in lion’s mane and maitake mushrooms, and very low levels were detected in oyster and shiitake mushrooms. Tryptophan was also measured in much lower concentrations in the specialty mushrooms (lion’s mane, maitake, oyster, shiitake) compared to *A. bisporus* mushroom varieties. Mushrooms have also been regarded as a rich source of the antioxidant glutathione, with previous research reporting levels ranging from 0.11 mg/g dry weight in *Cantharellus cibarius* (chanterelle) to 2.41 mg/g dry weight in *G. frondose* (maitake) [51]. We confirmed levels of glutathione were highest in *G. frondose*, compared to other mushroom varieties. In contrast to the previously cited work, glutathione levels in *H. erinaceus* were the lowest among the mushroom varieties. Nonetheless, this work supports the idea that mushrooms may be an important source of this dietary antioxidant. Notably, the concentration of amino acids does not account for digestibility or bioavailability, which must be considered when evaluating overall protein quality. Crudely, our findings are in line with previous work, which reports the limiting amino acids for several mushroom species, including oyster and shiitake, are lysine, methionine, and/or tryptophan [50]. Consistent with the results from the untargeted metabolomics analysis, the amino acid profiles vary among mushroom varieties, further differentiating the nutritional properties of different mushrooms.

We confirmed that lion’s mane and oyster mushrooms are among the best sources of the diet-derived amino acid, L-ergothioneine (Figure 8, Supplemental File S4). The variation of L-ergothioneine within some mushroom varieties in our study may be explained by differences in cultivation, handling, or degradation rates. To understand such variability, it will be necessary to conduct extensive stability studies, which would be of value to consumers, but may be challenging due to limitations such as harvest and shipping time. It is also noteworthy that similar differences in L-ergothioneine concentration have been reported on the USDA FoodData Central Database (https://fdc.nal.usda.gov/, accessed on 31 July 2022) for all seven mushrooms. For example, among eight analytical samples, L-ergothioneine concentrations ranged from 4 to 29 mg/100 g and 7 to 46 mg/100 g in oyster and lion’s mane mushrooms, respectively [35,36]. Taken together, these data suggest region or farming practices/cultivation conditions may influence L-ergothioneine concentrations. Future research may aim to measure L-ergothioneine concentrations of mushrooms from various geographic regions and under different cultivation conditions to better understand its impact.

As discussed here, mushrooms are a unique dietary source with several essential nutrients (described in the introduction) and a multitude of bioactive compounds. Despite the distinct properties of mushrooms from plant and animal food sources, mushrooms are currently categorized as an “other” vegetable by the Dietary Guidelines for Americans (DGA) [52]. As such, mushrooms are listed among approximately 30 “other” vegetables, which may underscore their importance as a functional food. The addition of a third food kingdom, “fungi/mycology”, was recently proposed, which may increase the recognition of mushrooms as a nutritionally unique food [1]. To expand on this, the detection of tens to hundreds of unique-to-mushroom-variety compounds from our work highlights areas for future research, particularly on the effects of consuming specialty mushrooms (i.e., lion’s mane, oyster, shiitake, maitake) on human health outcomes. Continued work in this area may further emphasize the need to differentiate mushrooms from plant foods and create subgroups (similar to the vegetable subgroups in the DGA) with recommendations for different varieties and amounts of mushrooms, as supported by robust experimental research.

A considerable amount of this discussion focuses on interesting (i.e., potential mushroom-specific, bioactive, etc.) compounds detected in our mushroom samples and potential health benefits [33,37,38,39,40,42]. As previously mentioned, evidence to support these potential health impacts is based on isolated compounds from mushrooms and not on the consumption of whole, dietary mushrooms. Furthermore, most of the health properties described herein have been demonstrated in cell and animal models. Thus, caution should be taken when interpreting our findings for translation to the public. To reiterate from above, high-quality experimental research is needed to: (1) assess the concentration of bioactive compounds in commercially available whole, fresh mushrooms, (2) determine compound bioavailability (absorption and retention/utilization) in humans, (3) investigate the potential utility as a biomarker of intake, and (4) evaluate the effects of acute and chronic consumption on human health outcomes.

Limitations of this study primarily relate to the inherent limitations of untargeted metabolomics analysis. The compounds detected and described here are putative, based on spectral data, and correspond to an MSI level three; unfortunately, obtaining informative MS/MS spectra was beyond the scope of the current study. The compounds are also in relative concentrations to the other mushroom samples. While this work is suitable for generating hypotheses for future research, the true quantitation of compounds will require targeted metabolomics analysis, which is both time-consuming and expensive. Another limitation is the lack of information available in metabolomics databases. Approximately 70% (3036/4255) of the compounds in sample replicates in this analysis were not annotated (i.e., named and identifiable in metabolomics databases) at the time of the study, highlighting areas for growth in the metabolomics field. Similarly, compound curation is a highly manual process that is only as informative as the available data/literature on a given compound. While many of the compounds described here suggest potential health benefits, there are a myriad of compounds in which even basic information, such as compound taxonomy, was unavailable. These databases are continually being updated, so the information discussed here reflects what was available at the time of the study. We employed a stringent filter when studying the compounds detected in the seven mushroom varieties to improve the confidence of the compound being endogenous to the mushroom(s). This may have filtered out other relevant compounds that were not detected in sample replicates and, therefore, were not included in this analysis. Finally, it is known that the chemical composition of foods, including some bioactive compounds, can vary depending on cultivation methods, including available nutrients, harvest time, harvest method, etc. While we were not able to obtain this information, results indicate that differences among mushroom subtypes were more due to the subtype than due to the farm. If cultivation methods had been responsible for differences among subtypes, then clustering in Figure 3 would have been by farm and not subtype.

## 5. Conclusions

This exploratory research confirms that mushrooms may be considered a functional food, capable of delivering many bioactive compounds beyond the traditional macro- and micronutrients that may promote health with routine consumption. While the 1344 compounds in common among the seven mushroom varieties support some level of similarity, the detection of hundreds of unique-to-mushroom-variety compounds and differences in amino acid profiles indicate that not all mushrooms are chemically comparable. Given the detection of >400 unique-to-mushroom-variety compounds in lion’s mane, maitake, oyster, and shiitake mushrooms, we suggest further targeted investigations on the compounds detected and potential health benefits. This work also highlights the usefulness of using an untargeted metabolomics approach to determine differences and similarities in chemical composition among mushroom varieties, identify potential mushroom-specific compounds that may serve as biomarkers of consumption, and lay the groundwork for linking these compounds to health benefits.

## Figures and Tables

**Table 1:** Summary of mushroom varieties and farm sourcing.

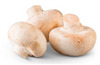		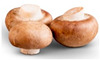	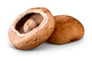	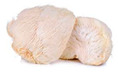	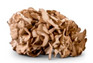	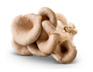	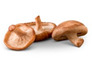
**White Button**		**Crimini**	**Portabella**	**Lion’s Mane**	**Maitake**	**Oyster**	**Shiitake**
***A. bisporus***		***A. bisporus***	***A. bisporus***	***H. erinaceus***	***G. frondose***	***P. ostreatu*** ***s***	***L. edodes***
Farm 1:	A	A	A	A	A	A	A
Farm 2:	B	B	B	C	C	C	C
Reps/Farm:	3	3	3	3	3	3	3
Total Reps:	6	6	6	6	6	6	6

Abbreviations: Reps—replicates

**Table 2:** Summary of compounds detected in sample replicates of any of the seven mushroom varieties that are significantly different.

Mushroom Variety	Crimini	Lion’s Mane	Maitake	Oyster	Portabella	Shiitake	White Button
**Crimini**	5690 *	**2327**	**2487**	**2485**	**255**	**1974**	**285**
**Lion’s Mane**	3363	5690	**2312**	**2176**	**2398**	**2086**	**2202**
**Maitake**	3203	3378	5690	**1969**	**2571**	**2126**	**2313**
**Oyster**	3205	3514	3721	5690	**2547**	**2096**	**2349**
**Portabella**	5435	3292	3119	3143	5690	**2096**	**411**
**Shiitake**	3716	3604	3564	3594	3594	5690	**1856**
**White button**	5405	3488	3377	3341	5279	3834	5690

* Includes hydrophobic and hydrophilic fractions. Data are the total number of compounds detected in sample replicates of any mushroom variety that passed the ANOVA thresholds (*p* < 0.05) with Benjamini–Hochberg multiple testing correction. That is, 5690 compounds detected in four of six sample replicates of any of the seven mushroom varieties passed the ANOVA thresholds (gold boxes). Bolded values in the light yellow boxes are the number of compounds that are significantly different between different mushroom varieties (i.e., 2327 compounds are significantly different between crimini and lion’s mane mushrooms). Values in the gray boxes are not significantly different between mushroom varieties.

**Table 3:** Summary of compounds detected in sample replicates of all seven mushroom varieties that are significantly different.

Mushroom Variety	Crimini	Lion’s Mane	Maitake	Oyster	Portabella	Shiitake	White Button
**Crimini**	549 *	**212**	**216**	**194**	**49**	**212**	**22**
**Lion’s Mane**	337	549	**200**	**175**	**232**	**194**	**223**
**Maitake**	333	349	549	**177**	**247**	**190**	**224**
**Oyster**	355	374	372	549	**224**	**185**	**189**
**Portabella**	500	317	302	325	549	**220**	**38**
**Shiitake**	337	355	359	364	329	549	**194**
**White button**	527	326	325	360	511	355	549

* Includes hydrophobic and hydrophilic fractions. Data are the total number of compounds detected in sample replicates of all seven mushroom varieties that passed the ANOVA thresholds (*p < 0.05*) with Benjamini–Hochberg multiple testing correction. That is, 549 compounds detected in four of six sample replicates of all seven mushroom varieties passed the ANOVA thresholds (gold boxes). Bolded values in the light yellow boxes are the number of compounds that are significantly different between different mushroom varieties (i.e., 212 compounds are significantly different between crimini and lion’s mane mushrooms. Values in the gray boxes are not significantly different between mushroom varieties.

**Table 4:** Categorization of food-specific compounds.

Mushroom Variety	Total Compounds (Annotated) *	Previously Determined to be in that Food ^1^	Probably/ Possibly in that Food ^2^	Found in Some/Any Other Food	Natural Product ^3^	Other ^4^	Cannot Determine ^5^
Detected in all 7	520	0	113	259	11	18	119
UNIQUE TO:							
White button	13	0	5	4	0	2	2
Crimini	4	0	0	1	0	1	2
Portabella	13	0	1	5	0	2	5
Lion’s mane	211	11	20	60	7	25	88
Maitake	206	1	8	97	21	14	65
Oyster	126	8	4	56	10	9	39
Shiitake	126	8	9	46	0	3	60
Sum	1219	28	160	528	49	74	380

* Includes hydrophobic and hydrophilic fractions. Note: categorization of compounds was determined based on available information using The Human Metabolome Database (HMDB; https://hmdb.ca/, first accessed on 9 May 2022), FooDB (https://foodb.ca/, first accessed on 9 May 2022), Kyoto Encyclopedia of Genes and Genomes (KEGG; https://www.genome.jp/kegg/, first accessed on 9 May 2022), Lipid Maps (https://www.lipidmaps.org/, first accessed on 9 May 2022), Google, and PubMed (https://pubmed.ncbi.nlm.nih.gov/, first accessed on 9 May 2022). 1: found in that mushroom variety; 2: identified in mushrooms generally; 3: natural product, not known to be found in mushrooms or foods; 4: putatively annotated as exogenous, non-natural products; 5: not enough information available.

**Table 5:** Proposed potential mushroom-specific compounds detected in white button, oyster, or in all seven mushroom varieties.

Mushroom Variety	Categorization	Compound	Main Class	Subclass	Notes
White button	Probably/ possibly in that food	(3beta,5alpha,9alpha,22E,24R)-5,9-Epidioxy-3-hydroxyergosta-7,22-dien-6-one Esi + 13.774996	Prenol Lipids	Sesquiterpenoids	HMDB: found in common and oyster mushrooms. Constituent of Hypsizygus marmoreus (bunashimeji) and Pleurotus ostreatus (oyster mushroom).
		Ergosterol peroxide Esi + 13.684002	Steroids and Steroid Derivatives	Ergostane Steroids	Ergosterol peroxide is a secondary metabolite commonly detected in different mushrooms [33,34].
Oyster	Previously determined to be in that food	(3beta,5alpha,9alpha,22E,24R)-5,9-Epidioxy-3-hydroxyergosta-7,22-dien-6-one	Prenol Lipids	Sesquiterpenoids	HMDB: Constituent of *Hypsizygus marmoreus* (bunashimeji) and *Pleurotus ostreatus* (oyster mushroom).
		2-Acetoxy-3-geranylgeranyl-1,4-dihydroxybenzene	Prenol Lipids	Diterpenoids	HMDB: found in common and oyster mushrooms. FooDB: associated with common and oyster mushrooms.
		Methyl (Z,Z)-10-hydroxy-2,8-decadiene-4,6-diynoate	Fatty Acyls	Fatty Alcohols	HMDB: found in common and oyster mushrooms. FooDB: associated with common and oyster mushrooms.
	Probably/ possibly in that food	Polyporusterone E Esi + 0.83	Steroids and Steroid Derivatives	Cholestane Steroids	HMDB: found in common and oyster mushrooms FooDB: associated with common and oyster mushrooms.
All 7 varieties	Probably/ possibly in that food	Cerebroside B	Prenol Lipids	Triterpenoids	FoodDB: associated with common and oyster mushrooms.
		N-(2R-Hydroxyhexadecanoyl)-2S-amino-9-methyl-4E,8E-octadecadiene-1,3R-diol Esi + 4.5509977	Sphingolipids	Ceramides	HMDB: found in common and oyster mushrooms. FooDB: associated with common and oyster mushrooms.
		(3beta,22E,24R)-Ergosta-4,6,8(14),22-tetraen-3-ol Esi + 15.40999	Steroids and Steroid Derivatives	Ergostane Steroids	FoodDB: associated with common and oyster mushrooms.

Abbreviations: HMDB—The human metabolome database (https://hmdb.ca/, first accessed on 9 May 2022); FooDB—The food database (https://foodb.ca/, first accessed on 9 May 2022).

## Data Availability

The data for the 1219 compounds presented in this study are available in the article and Supplementary Material. Requests for data on other compounds should be made to the corresponding author.

## Supplementary Materials

The following supporting information can be downloaded at: https://www.mdpi.com/article/10.3390/foods12162985/s1, File S1: Compounds detected in sample replicates (detected in all 7 and unique to mushroom variety); File S2: amino acid profiles of the seven different mushroom varieties; File S3: Compounds that passed ANOVA thresholds (detected in all 7 mushrooms only); File S4: Amino acid ANOVAs; File S5: Supplemental Figure S1 (Select bioactive compounds detected in sample replicates of seven mushroom varieties), Figure S2 (Principal Component Analysis (PCA) using data from the hydrophobic fraction of seven mushroom varieties along with the pooled instrument QCs), Figure S3 (Principal Component Analysis (PCA) using data from the hydrophilic fraction of seven mushroom varieties along with the pooled instrument QCs).
