# Supplementary material for: Metabolomics Profiling of White Button, Crimini, Portabella, Lion’s Mane, Maitake, Oyster, and Shiitake Mushrooms Using Untargeted Metabolomics and Targeted Amino Acid Analysis
Source: Foods. 2023 Aug 8;12(16):2985. doi: 10.3390/foods12162985 (PMC10453450; doi:10.3390/foods12162985)
Supplement: Supplementary file 1 [file foods-12-02985-s001.zip › File S4. Amino Acid ANOVAs.pdf]

Supplementary Material – Amino Acid ANOVAs

What's in a Mushroom? Dietary Mushroom Metabolomics Profiling Using Untargeted Metabolomics and Targeted Amino Acid Analysis

Cassi N Uffelman[1], Katrina A Doenges[2], Michael L Armstrong[2], Kevin Quinn[2], Minghua Tang[3], Nancy F Krebs[3], Nichole A Reisdorph[2], Wayne W Campbell[1]

Affiliations:

[1]Department of Nutrition Science, Purdue University, West Lafayette, IN 47907, USA.

[2] Skaggs School of Pharmacy and Pharmaceutical Sciences, University of Colorado Anschutz Medical Campus, Aurora, CO 80045, USA.

[3]School of Medicine, Department of Pediatrics, University of Colorado Anschutz Medical Campus, Aurora, CO 80045, USA.

Isoleucine

Mushroom Variety	Emmean	SE	df	Lower.CL	Upper.CL	Groups
Crimini	548.64	56.49	35	433.95	663.32	2
Lion's Mane	99.08	56.49	35	-15.60	213.77	1
Maitake	117.57	56.49	35	2.88	232.26	1
Oyster	75.35	56.49	35	-39.34	190.04	1
Portabella	473.20	56.49	35	358.51	587.89	2
Shiitake	171.00	56.49	35	56.31	285.68	1
White button	683.42	56.49	35	568.73	798.11	2

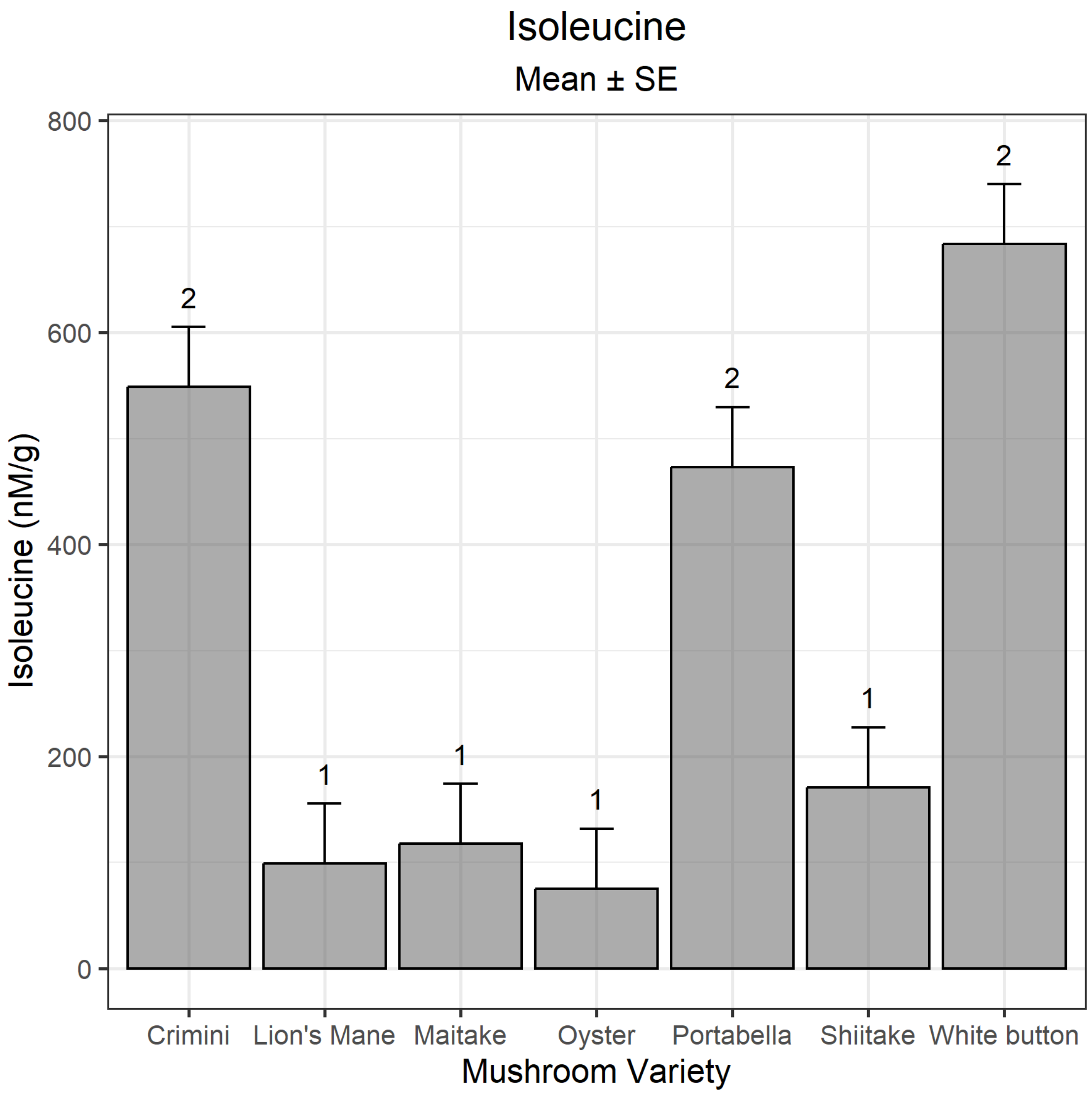

Data are pooled mean ± SE. Different numbers denote significance ($p < 0.05$).

Leucine

Mushroom Variety	Emmean	SE	df	Lower.CL	Upper.CL	Groups
Crimini	899.10	76.12	35	744.56	1053.64	2
Lion's Mane	288.86	76.12	35	134.32	443.40	1
Maitake	187.82	76.12	35	33.28	342.36	1
Oyster	199.01	76.12	35	44.47	353.55	1
Portabella	835.68	76.12	35	681.14	990.22	2
Shiitake	315.01	76.12	35	160.47	469.55	1
White button	1081.31	76.12	35	926.77	1235.85	2

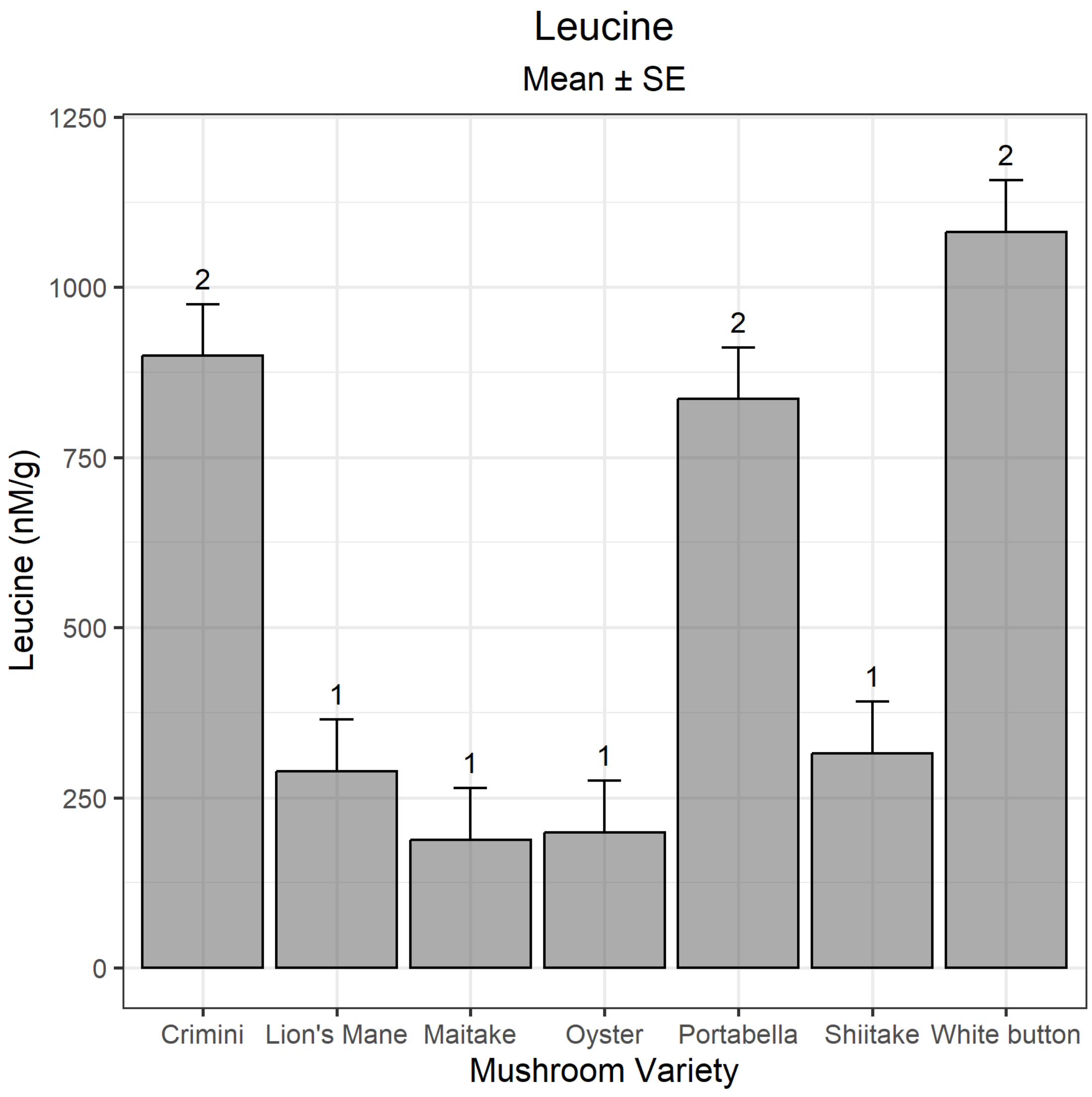

Data are pooled mean $\pm$ SE. Different numbers denote significance ($p < 0.05$).

Valine

Mushroom Variety	Emmean	SE	df	Lower.CL	Upper.CL	Groups
Crimini	773.37	71.90	35	627.41	919.34	3
Lion's Mane	230.50	71.90	35	84.54	376.46	1
Maitake	293.59	71.90	35	147.63	439.55	1
Oyster	177.40	71.90	35	31.44	323.36	1
Portabella	696.27	71.90	35	550.31	842.23	2,3
Shiitake	418.18	71.90	35	272.22	564.14	1,2
White button	969.06	71.90	35	823.10	1115.03	3

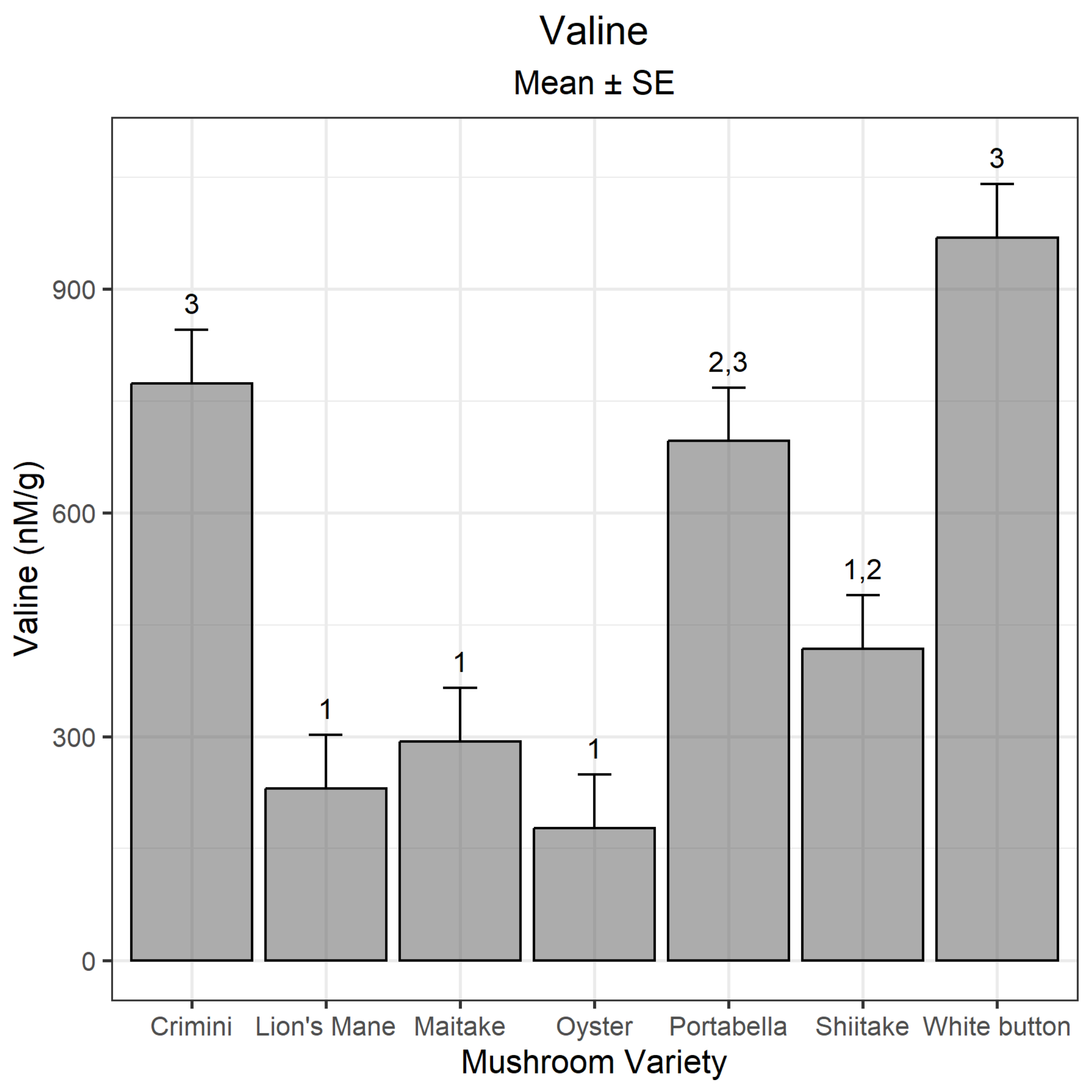

Data are pooled mean ± SE. Different numbers denote significance ($p < 0.05$).

Histidine

Mushroom Variety	Emmean	SE	df	Lower.CL	Upper.CL	Groups
Crimini	370.67	28.17	28	312.98	428.37	2
Lion's Mane	171.34	28.17	28	113.65	229.04	1
Maitake	213.82	28.17	28	156.12	271.51	1
Oyster	234.98	28.17	28	177.28	292.67	1
Portabella	270.60	28.17	28	212.90	328.29	1,2
Shiitake	175.41	28.17	28	117.72	233.11	1
White button	348.23	28.17	28	290.53	405.92	2

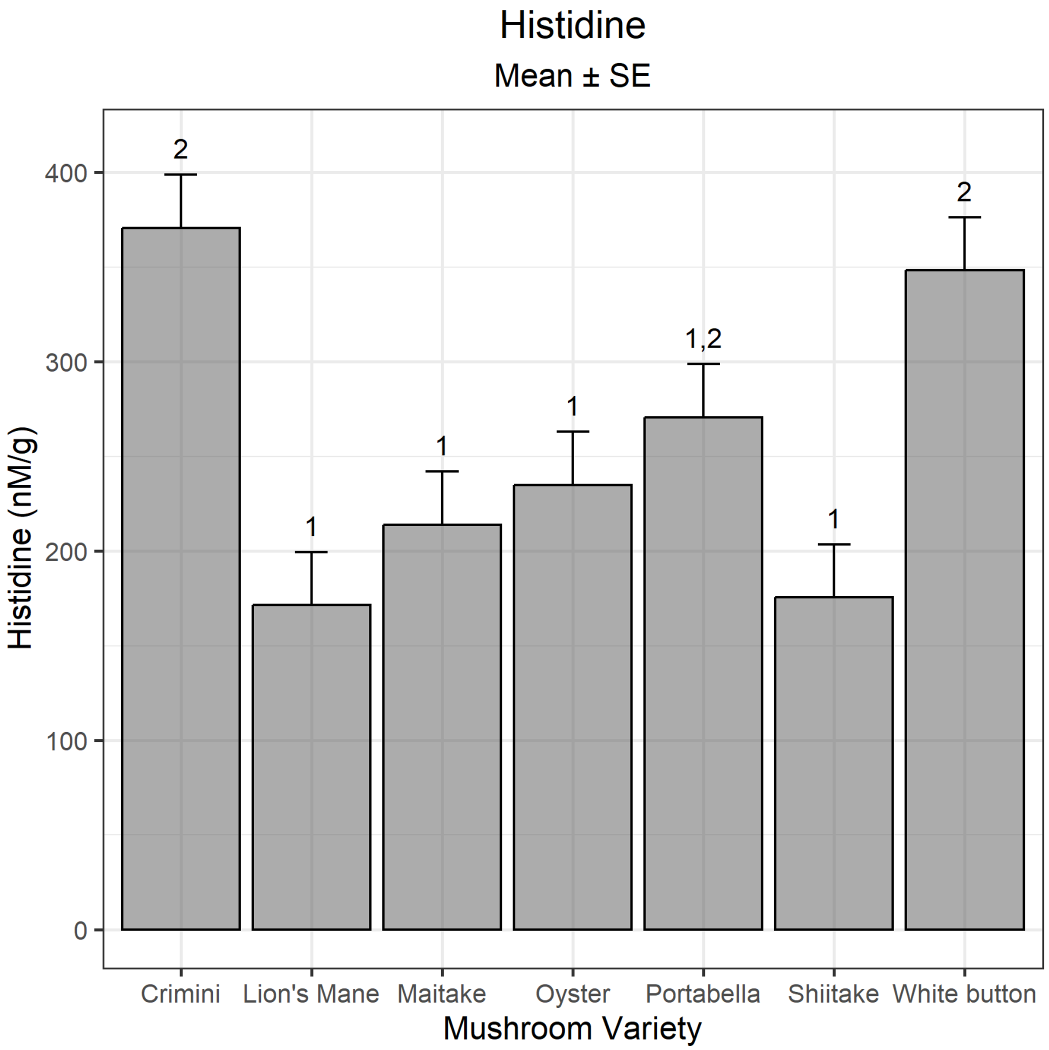

Data are pooled mean ± SE. Different numbers denote significance (p <0.05).

Lysine

Mushroom Variety	Emmean	SE	df	Lower.CL	Upper.CL	Groups
Crimini	288.51	44.15	28	198.06	378.95	1
Lion's Mane	340.57	44.15	28	250.12	431.01	1
Maitake	206.15	44.15	28	115.70	296.59	1,2
Oyster	81.34	44.15	28	-9.10	171.78	2
Portabella	282.99	44.15	28	192.55	373.44	1
Shiitake	345.20	44.15	28	254.76	435.65	1
White button	565.40	44.15	28	474.96	655.85	3

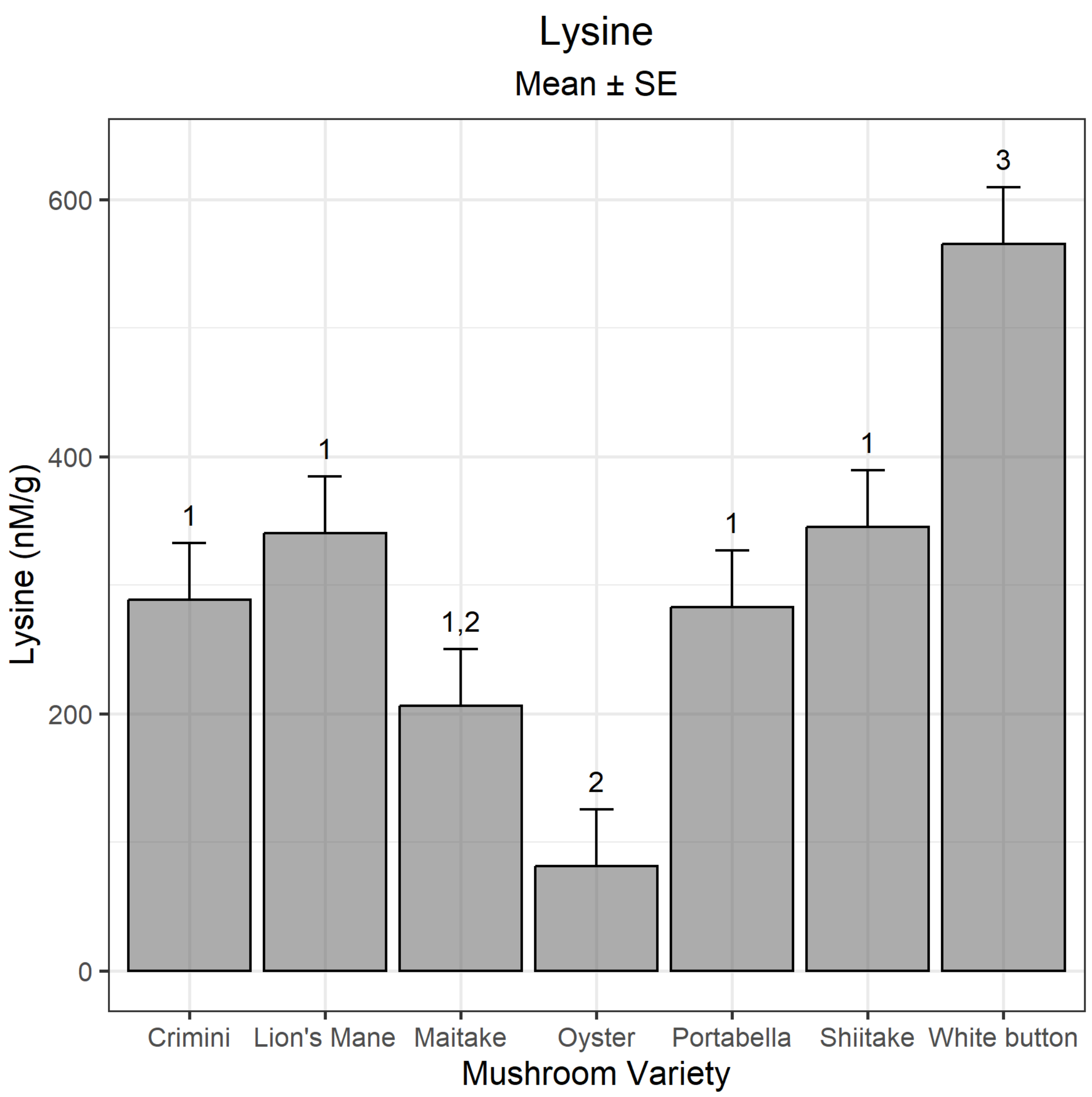

Data are pooled mean ± SE. Different numbers denote significance ($p < 0.05$).

Methioneine

Mushroom Variety	Emmean	SE	df	Lower.CL	Upper.CL	Groups
Crimini	145.41	20.79	35	103.19	187.63	2
Lion's Mane	0.00	20.79	35	-42.22	42.22	1
Maitake	0.00	20.79	35	-42.22	42.22	1
Oyster	4.69	20.79	35	-37.52	46.91	1
Portabella	142.11	20.79	35	99.90	184.33	2
Shiitake	5.80	20.79	35	-36.42	48.01	1
White button	209.92	20.79	35	167.70	252.13	2

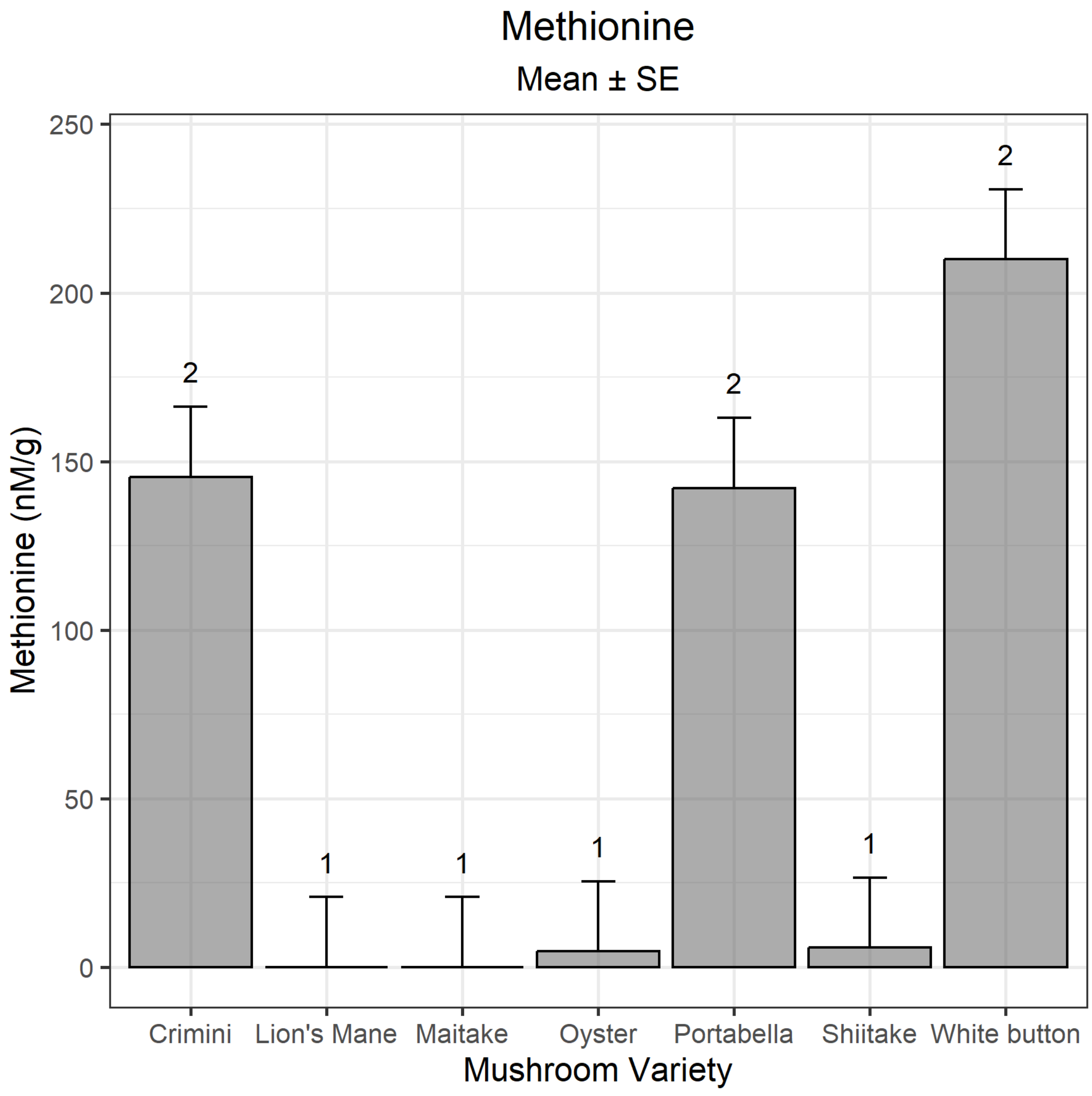

Data are pooled mean ± SE. Different numbers denote significance ($p < 0.05$).

Phenylalanine

Mushroom Variety	Emmean	SE	df	Lower.CL	Upper.CL	Groups
Crimini	566.79	36.75	33	492.03	641.55	3
Lion's Mane	82.30	37.19	33	6.64	157.96	1
Maitake	208.37	37.19	33	132.70	284.03	1,2
Oyster	293.49	37.19	33	217.83	369.15	2
Portabella	542.93	36.75	33	468.17	617.70	3
Shiitake	227.01	37.19	33	151.34	302.67	1,2
White button	632.56	36.75	33	557.80	707.32	3

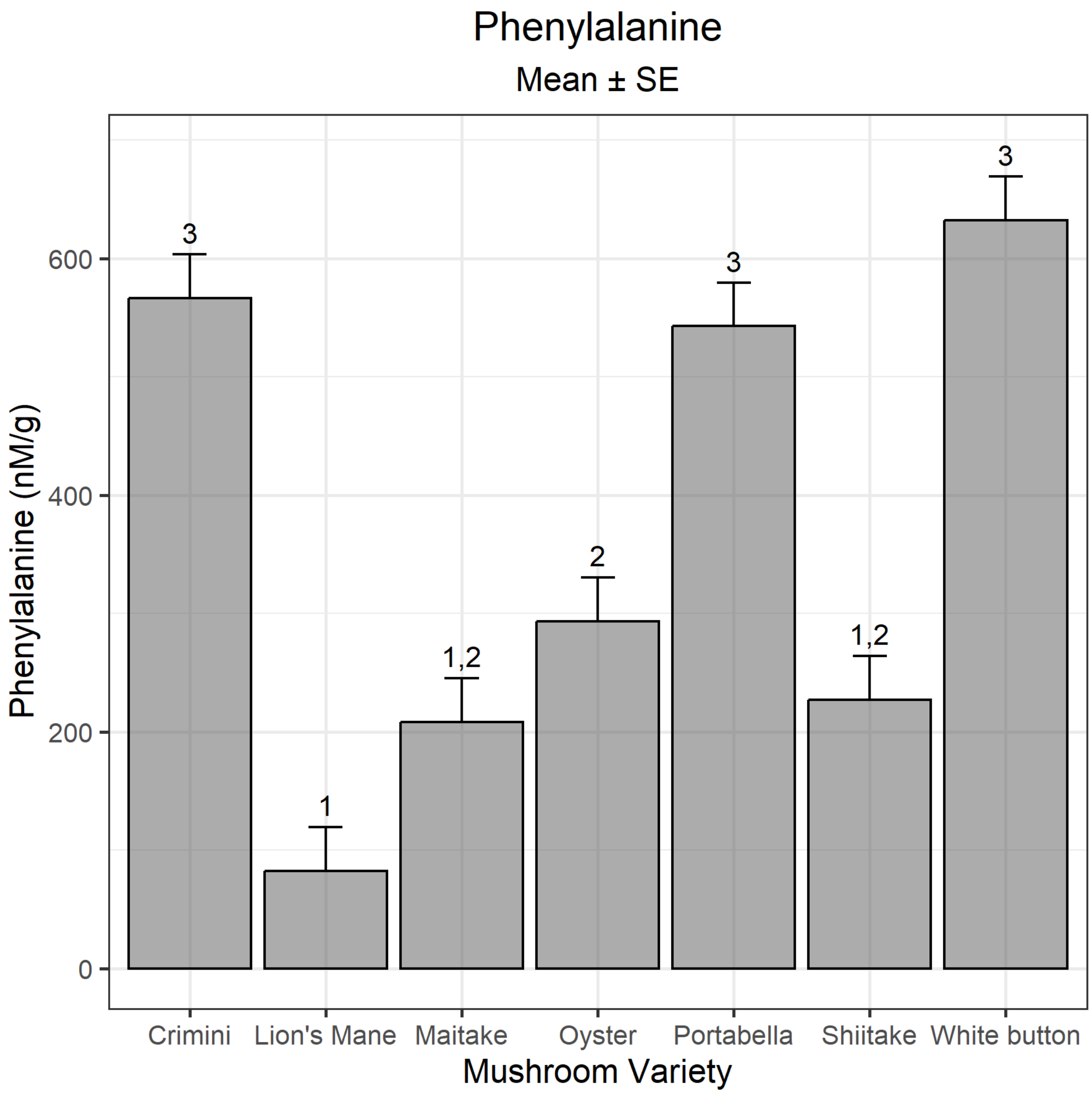

Data are pooled mean ± SE. Different numbers denote significance ($p < 0.05$).

Proline

Mushroom Variety	Emmean	SE	df	Lower.CL	Upper.CL	Groups
Crimini	282.88	55.62	35	169.96	395.80	1,2
Lion's Mane	153.44	55.62	35	40.52	266.36	1
Maitake	164.24	55.62	35	51.32	277.17	1
Oyster	91.15	55.62	35	-21.77	204.07	1
Portabella	506.82	55.62	35	393.90	619.75	2
Shiitake	167.66	55.62	35	54.73	280.58	1
White button	785.27	55.62	35	672.34	898.19	3

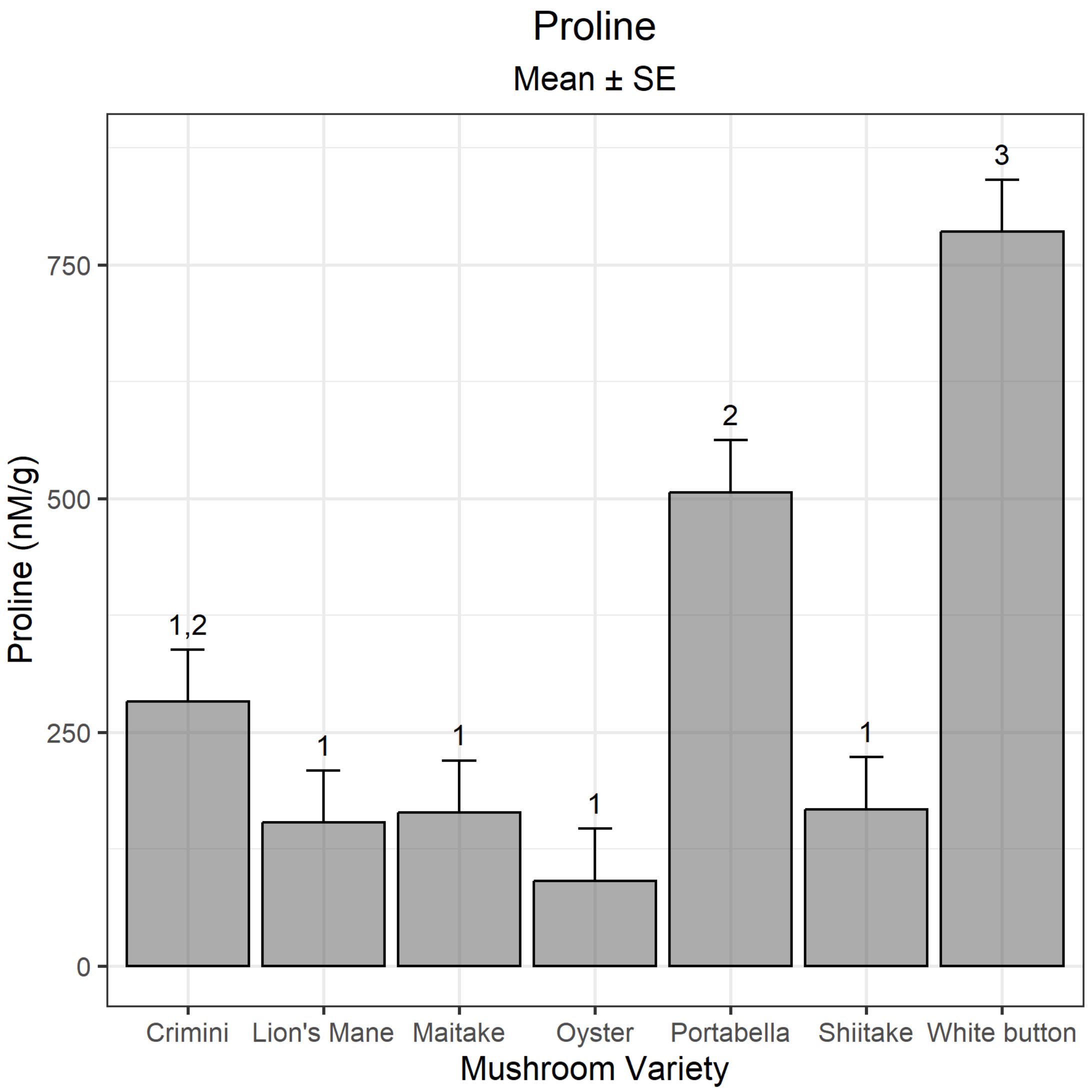

Data are pooled mean ± SE. Different numbers denote significance ($p < 0.05$).

Tryptophan

Mushroom Variety	Emmean	SE	df	Lower.CL	Upper.CL	Groups
Crimini	281.75	35.12	35	210.46	353.04	3
Lion's Mane	65.48	35.12	35	-5.81	136.78	1,2
Maitake	129.70	35.12	35	58.41	200.99	1,2
Oyster	77.15	35.12	35	5.86	148.44	1,2
Portabella	219.46	35.12	35	148.17	290.76	1,3
Shiitake	16.91	35.12	35	-54.38	88.20	2
White button	312.94	35.12	35	241.65	384.23	3

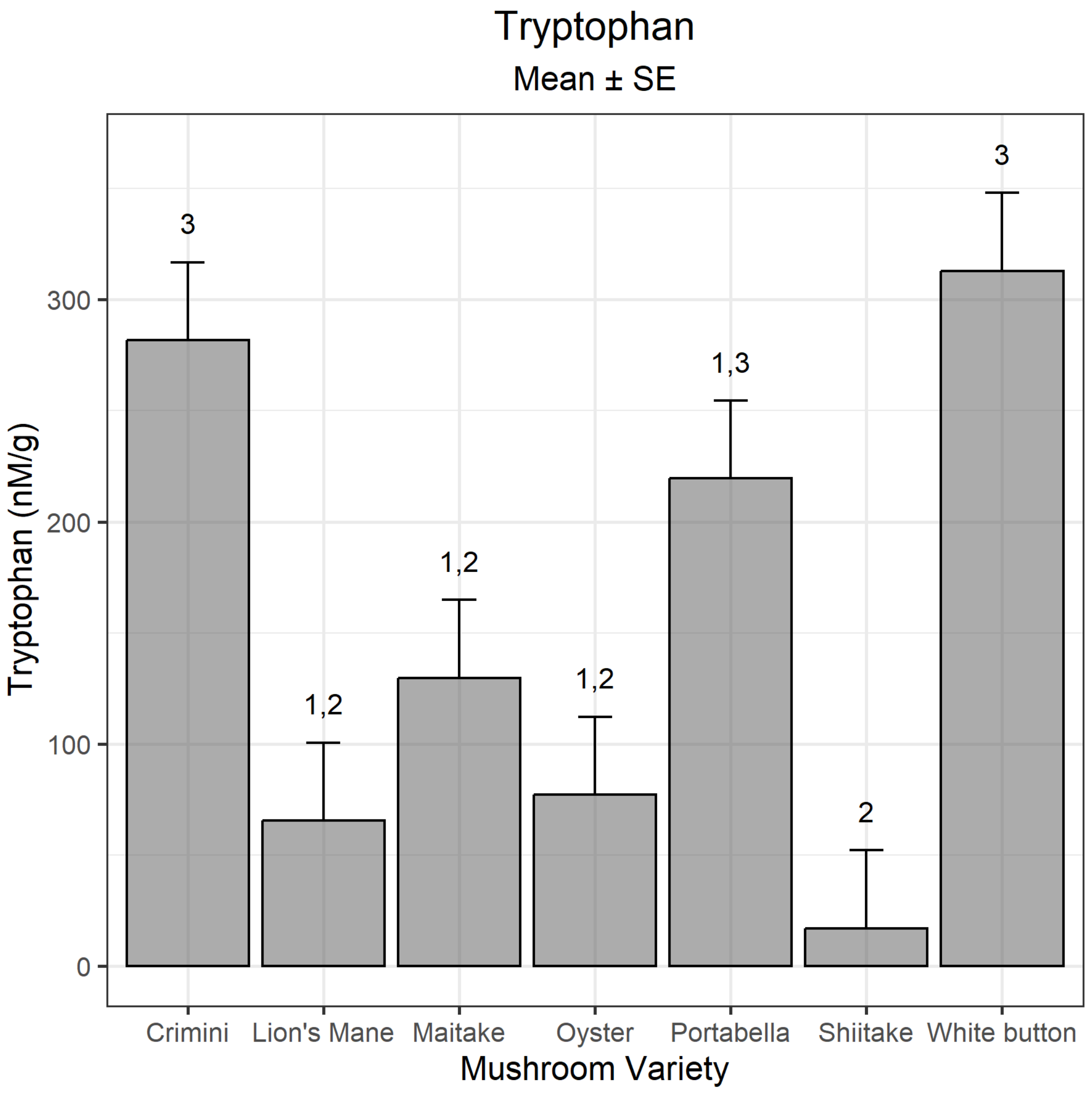

Data are pooled mean ± SE. Different numbers denote significance (p <0.05).

Alanine

Mushroom Variety	Emmean	SE	df	Lower.CL	Upper.CL	Groups
Crimini	1761.52	168.46	35	1419.53	2103.50	3
Lion's Mane	629.54	168.46	35	287.56	971.53	1
Maitake	315.37	168.46	35	-26.61	657.35	1
Oyster	593.44	168.46	35	251.46	935.42	1
Portabella	2093.64	168.46	35	1751.65	2435.62	2,3
Shiitake	726.17	168.46	35	384.19	1068.16	1
White button	2826.63	168.46	35	2484.65	3168.62	2

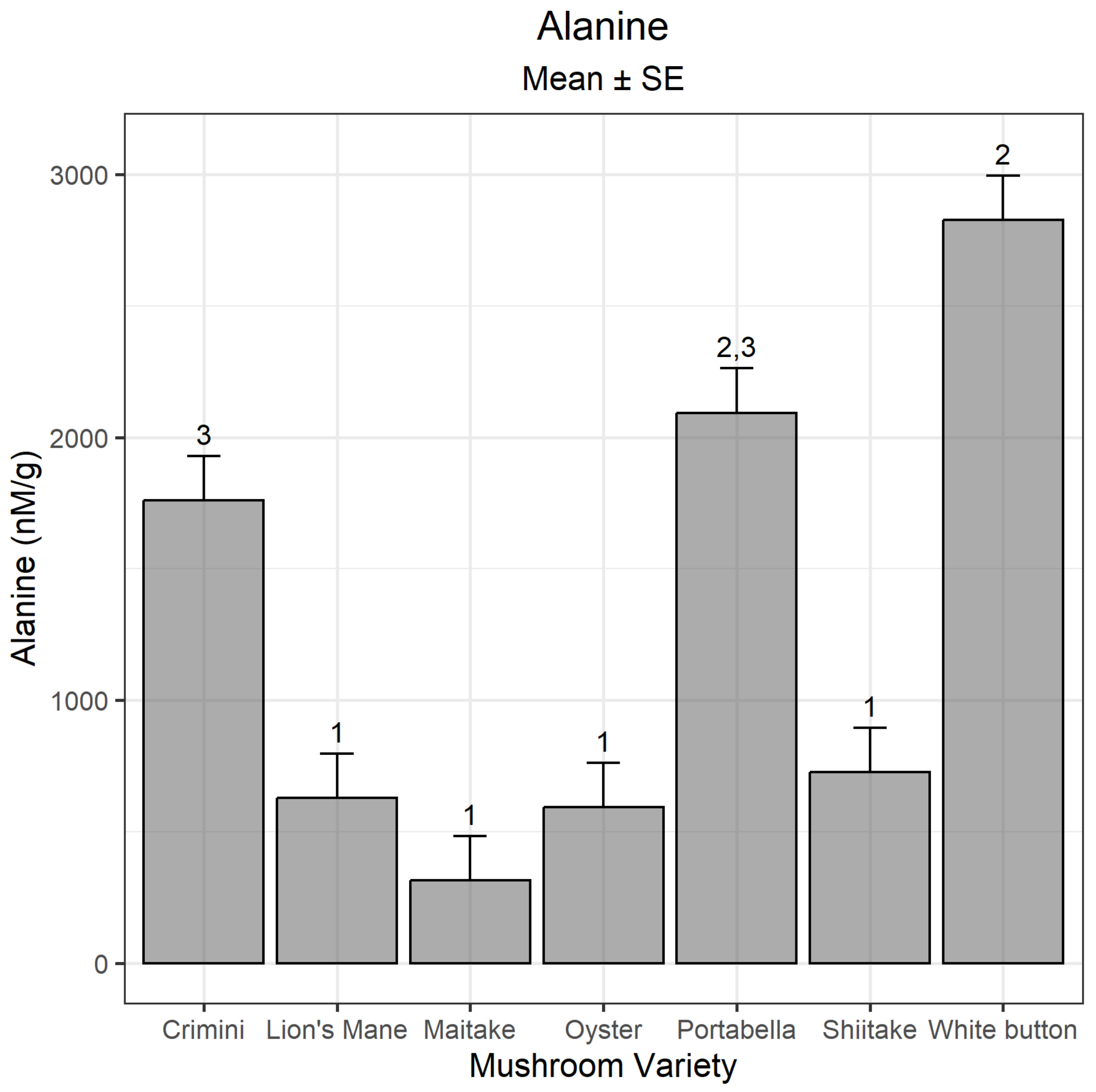

Data are pooled mean ± SE. Different numbers denote significance ($p < 0.05$).

Arginine

Mushroom Variety	Emmean	SE	df	Lower.CL	Upper.CL	Groups
Crimini	145.53	42.42	28	58.64	232.42	3
Lion's Mane	482.97	42.42	28	396.08	569.86	1
Maitake	236.16	42.42	28	149.27	323.05	2,3
Oyster	398.37	42.42	28	311.48	485.26	1,2
Portabella	124.46	42.42	28	37.57	211.35	3
Shiitake	215.64	42.42	28	128.75	302.53	2,3
White button	123.36	42.42	28	36.47	210.25	3

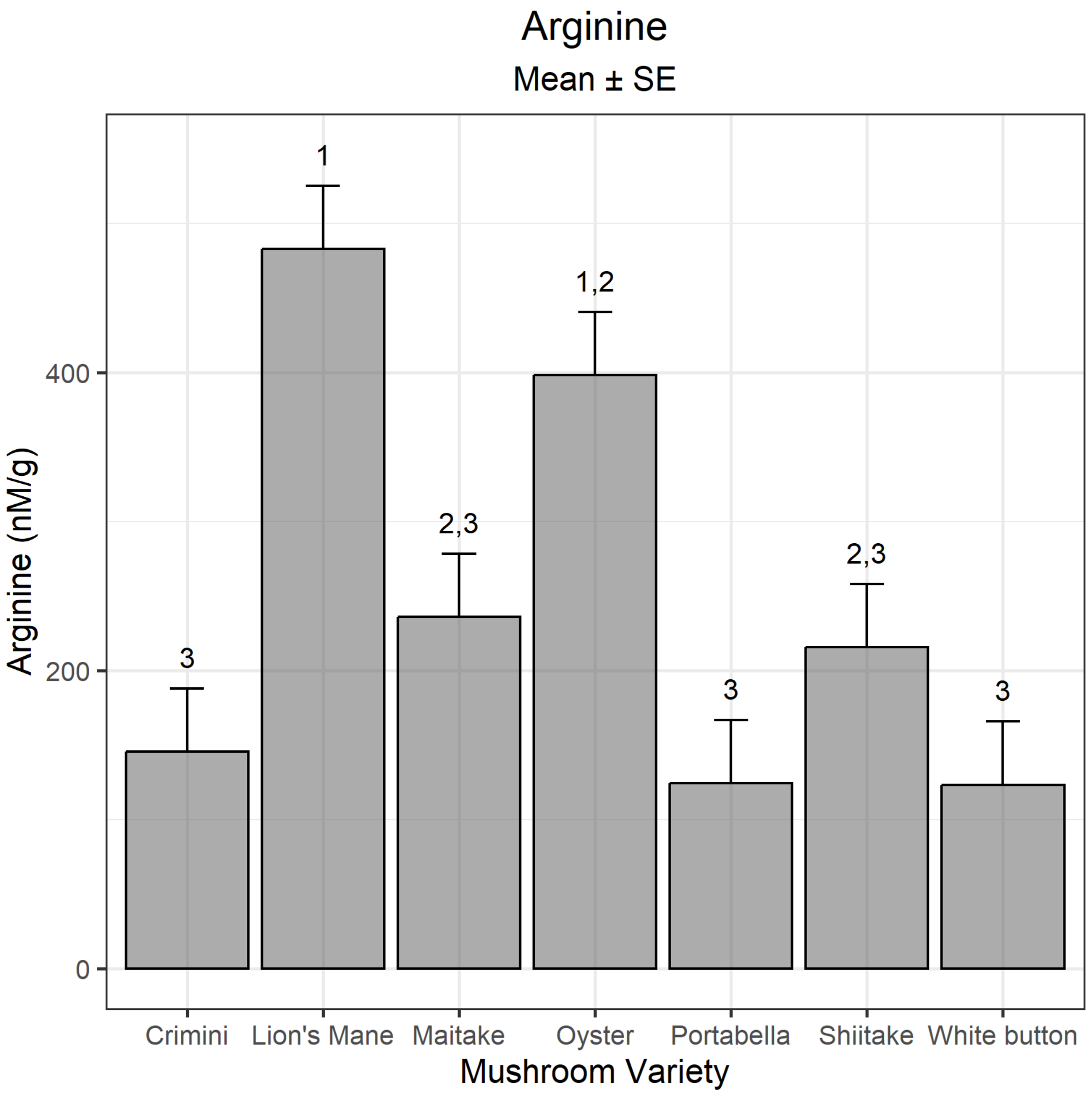

Data are pooled mean ± SE. Different numbers denote significance ($p < 0.05$).

Asparagine

Mushroom Variety	Emmean	SE	df	Lower.CL	Upper.CL	Groups
Crimini	1077.76	159.49	35	753.99	1401.54	2
Lion's Mane	289.49	159.49	35	-34.29	613.26	1
Maitake	173.47	159.49	35	-150.31	497.25	1
Oyster	337.83	159.49	35	14.05	661.60	1
Portabella	1084.45	159.49	35	760.68	1408.23	2
Shiitake	686.16	159.49	35	362.38	1009.94	1,2
White button	1990.72	159.49	35	1666.95	2314.50	3

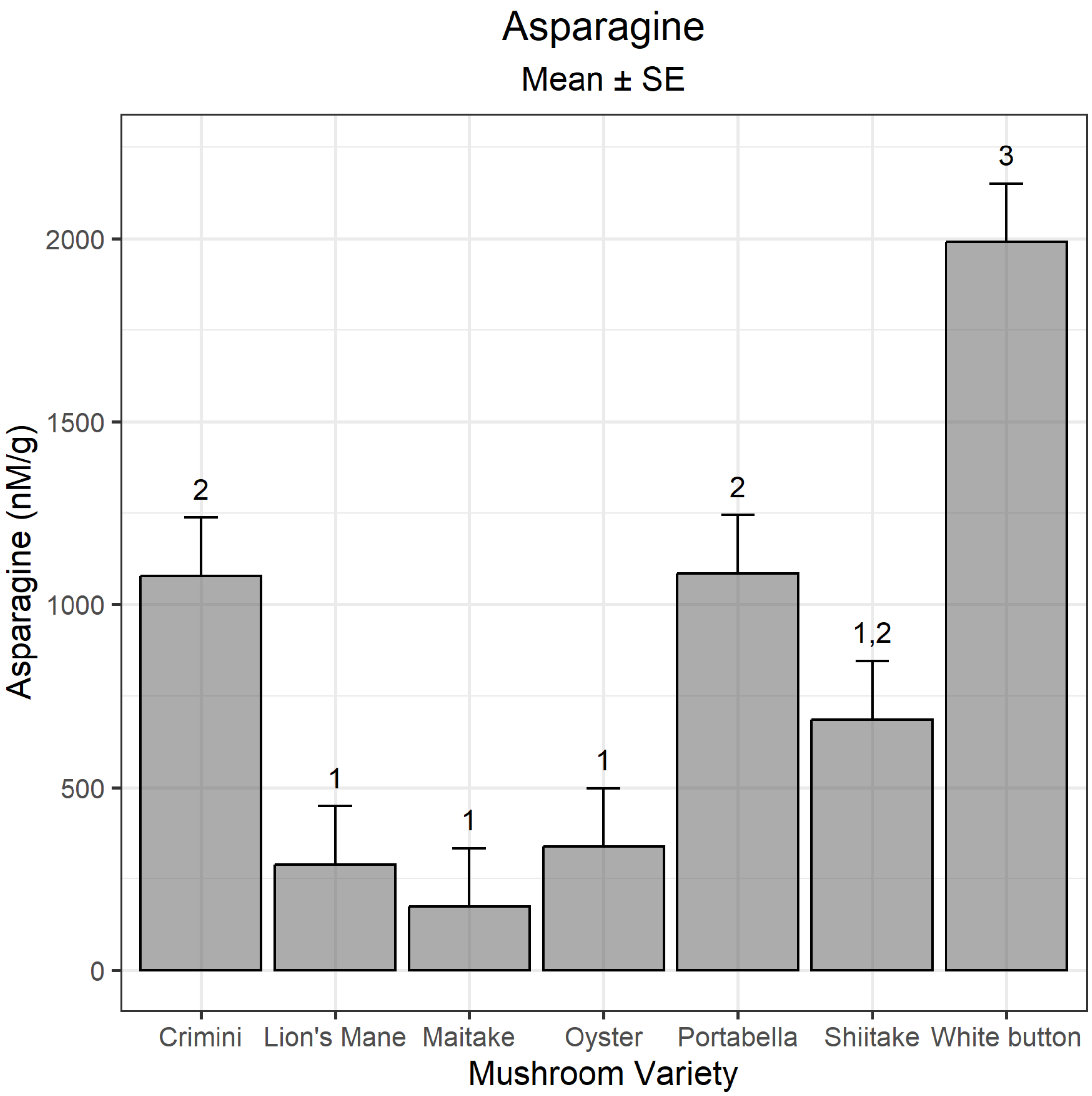

Data are pooled mean ± SE. Different numbers denote significance ($p < 0.05$).

Aspartic Acid

Mushroom Variety	Emmean	SE	df	Lower.CL	Upper.CL	Groups
Crimini	2145.60	145.06	28	1848.46	2442.74	2
Lion's Mane	1142.01	145.06	28	844.87	1439.16	1
Maitake	688.36	145.06	28	391.22	985.50	1
Oyster	1710.83	145.06	28	1413.69	2007.97	2
Portabella	1970.67	145.06	28	1673.53	2267.81	2
Shiitake	896.54	145.06	28	599.39	1193.68	1
White button	2343.77	145.06	28	2046.63	2640.92	2

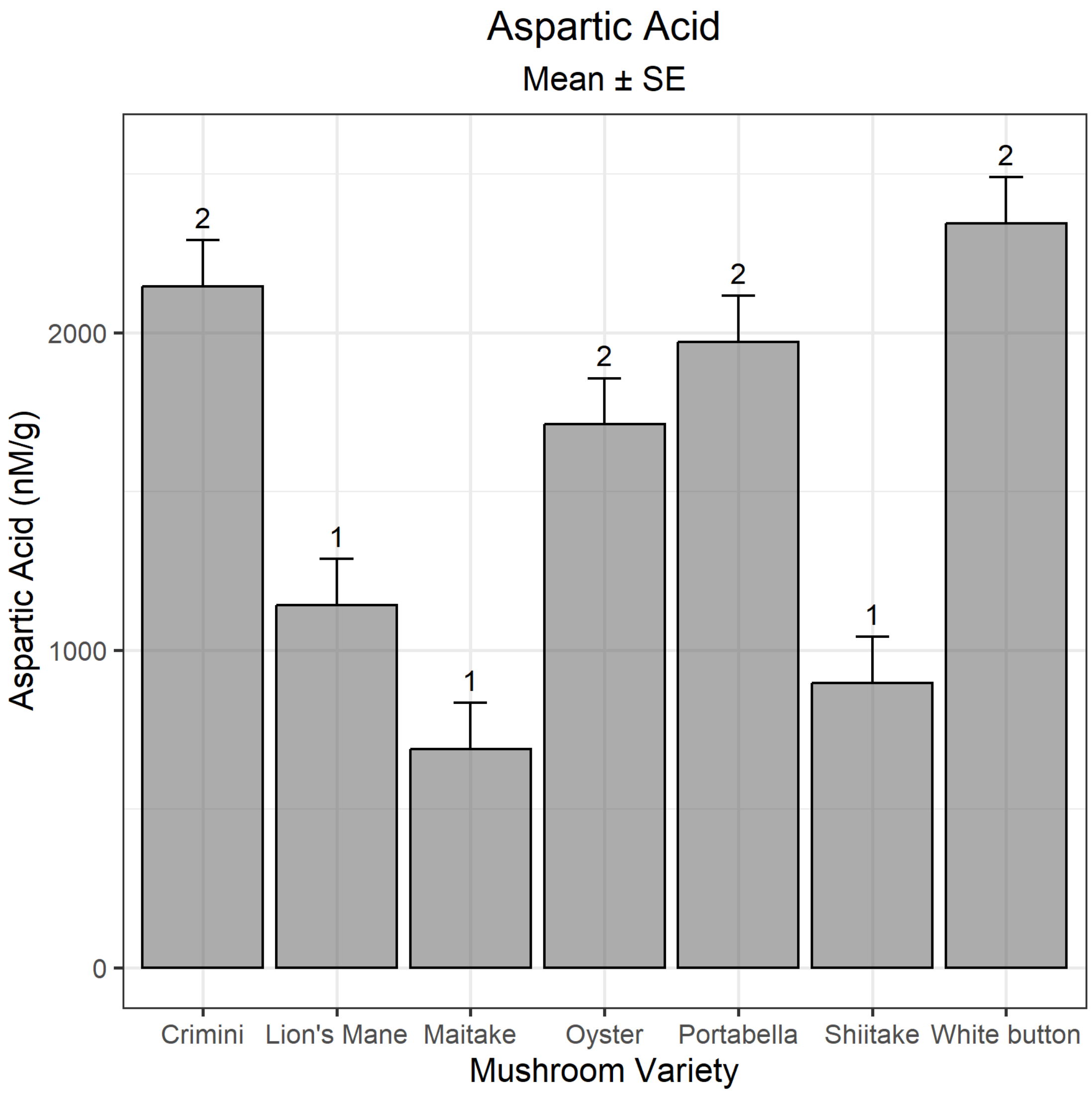

Data are pooled mean ± SE. Different numbers denote significance ($p < 0.05$).

Cysteine

Mushroom Variety	Emmean	SE	df	Lower.CL	Upper.CL	Groups
Crimini	997.07	107.56	35	778.71	1215.42	2
Lion's Mane	202.85	107.56	35	-15.51	421.20	1
Maitake	327.01	107.56	35	108.66	545.36	1
Oyster	357.17	107.56	35	138.81	575.52	1
Portabella	853.03	107.56	35	634.68	1071.39	2
Shiitake	731.74	107.56	35	513.38	950.09	2
White button	1092.58	107.56	35	874.23	1310.93	2

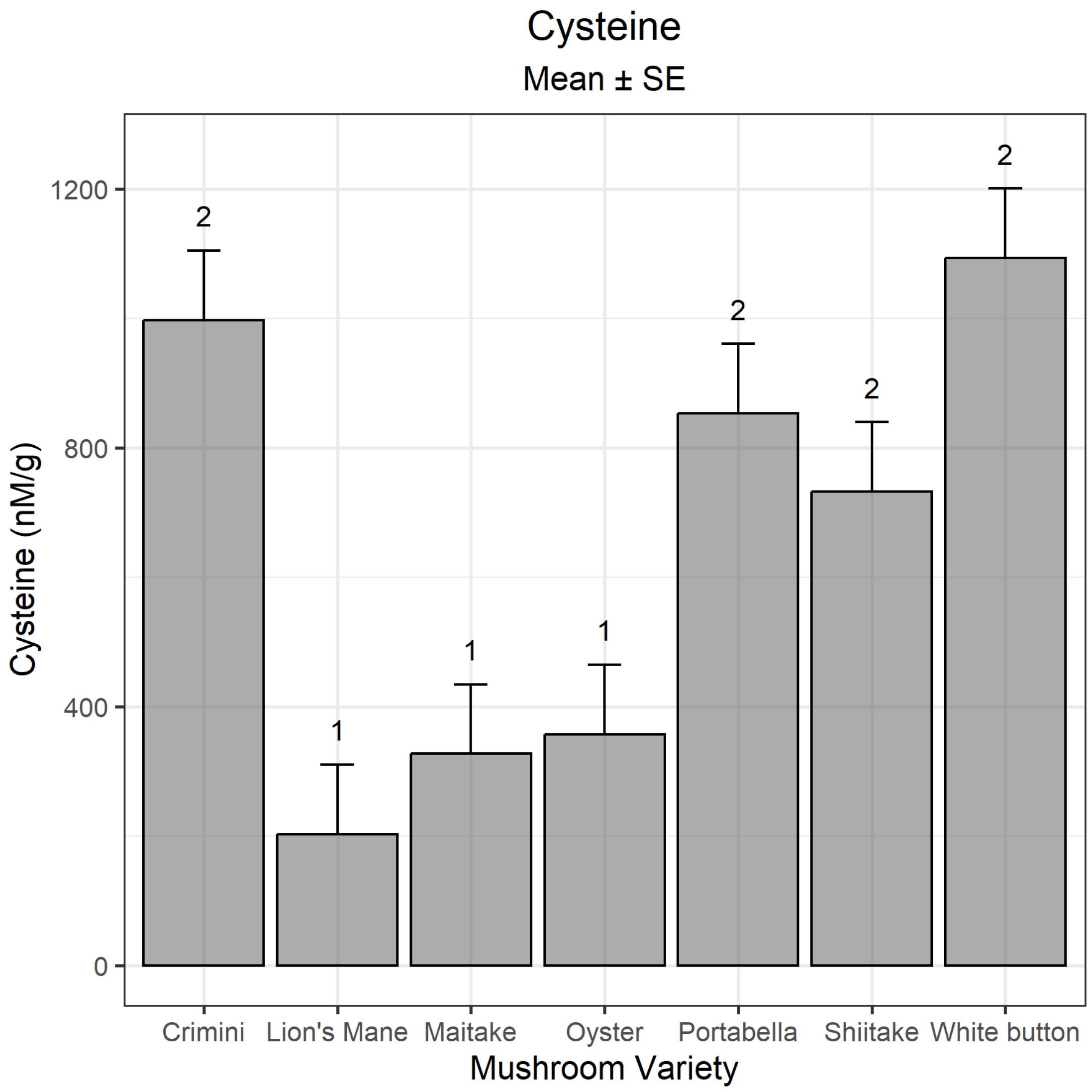

Data are pooled mean ± SE. Different numbers denote significance ($p < 0.05$).

Glutamic Acid

Mushroom Variety	Emmean	SE	df	Lower.CL	Upper.CL	Groups
Crimini	3194.50	282.79	33	2619.15	3769.85	1,3
Lion's Mane	2620.54	286.19	33	2038.27	3202.80	1
Maitake	1248.31	286.19	33	666.04	1830.58	2
Oyster	2705.84	286.19	33	2123.57	3288.10	1
Portabella	3971.37	282.79	33	3396.02	4546.72	3
Shiitake	1533.10	286.19	33	950.84	2115.37	2
White button	3713.80	282.79	33	3138.45	4289.15	3

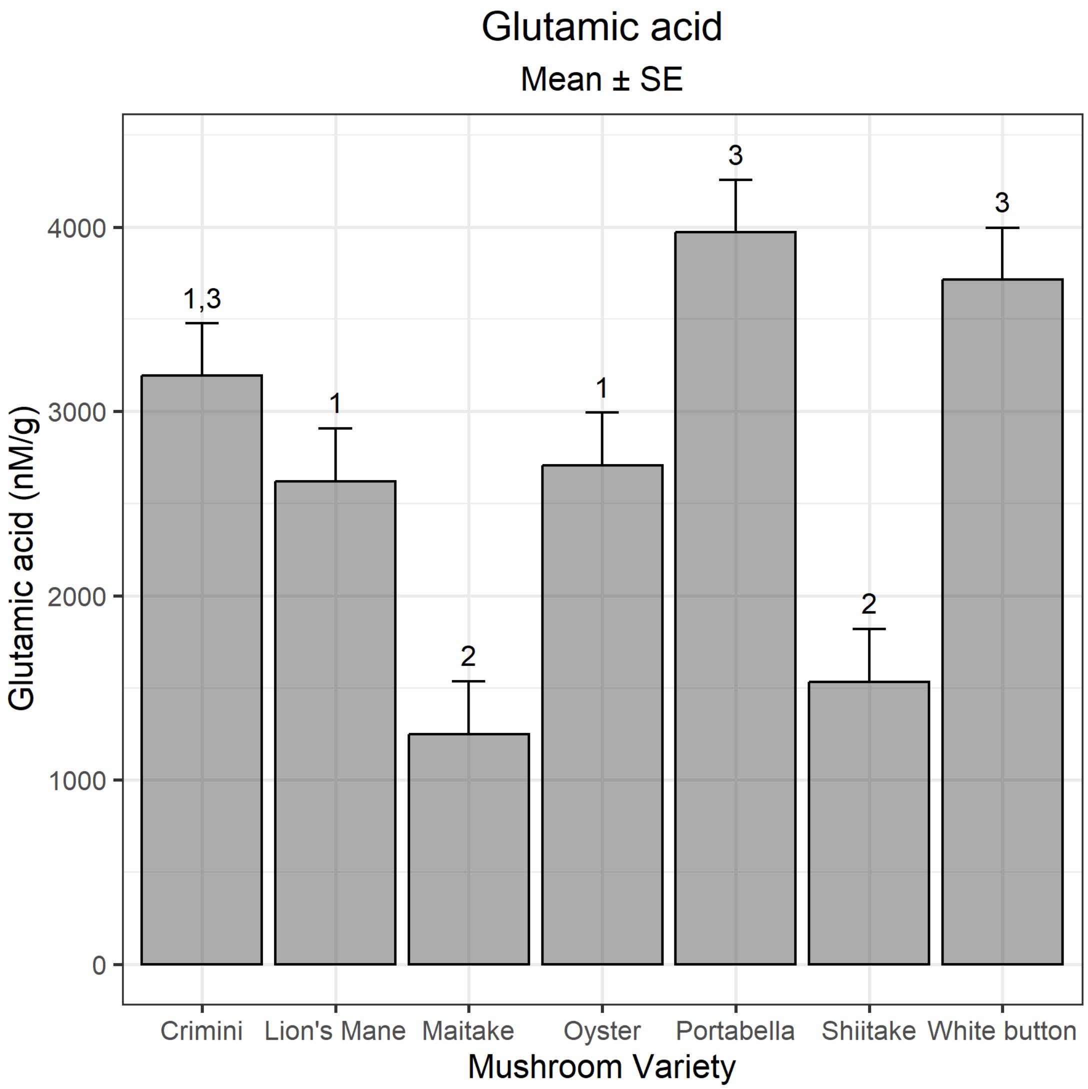

Data are pooled mean ± SE. Different numbers denote significance ($p < 0.05$).

Glutamine

Mushroom Variety	Emmean	SE	df	Lower.CL	Upper.CL	Groups
Crimini	2952.19	417.67	28	2096.64	3807.74	1,2
Lion's Mane	3739.02	417.67	28	2883.47	4594.57	1,3
Maitake	1172.66	417.67	28	317.11	2028.21	2
Oyster	1803.19	417.67	28	947.64	2658.74	2
Portabella	2641.07	417.67	28	1785.52	3496.62	1,2
Shiitake	5403.08	417.67	28	4547.53	6258.63	3
White button	4737.83	417.67	28	3882.28	5593.38	3

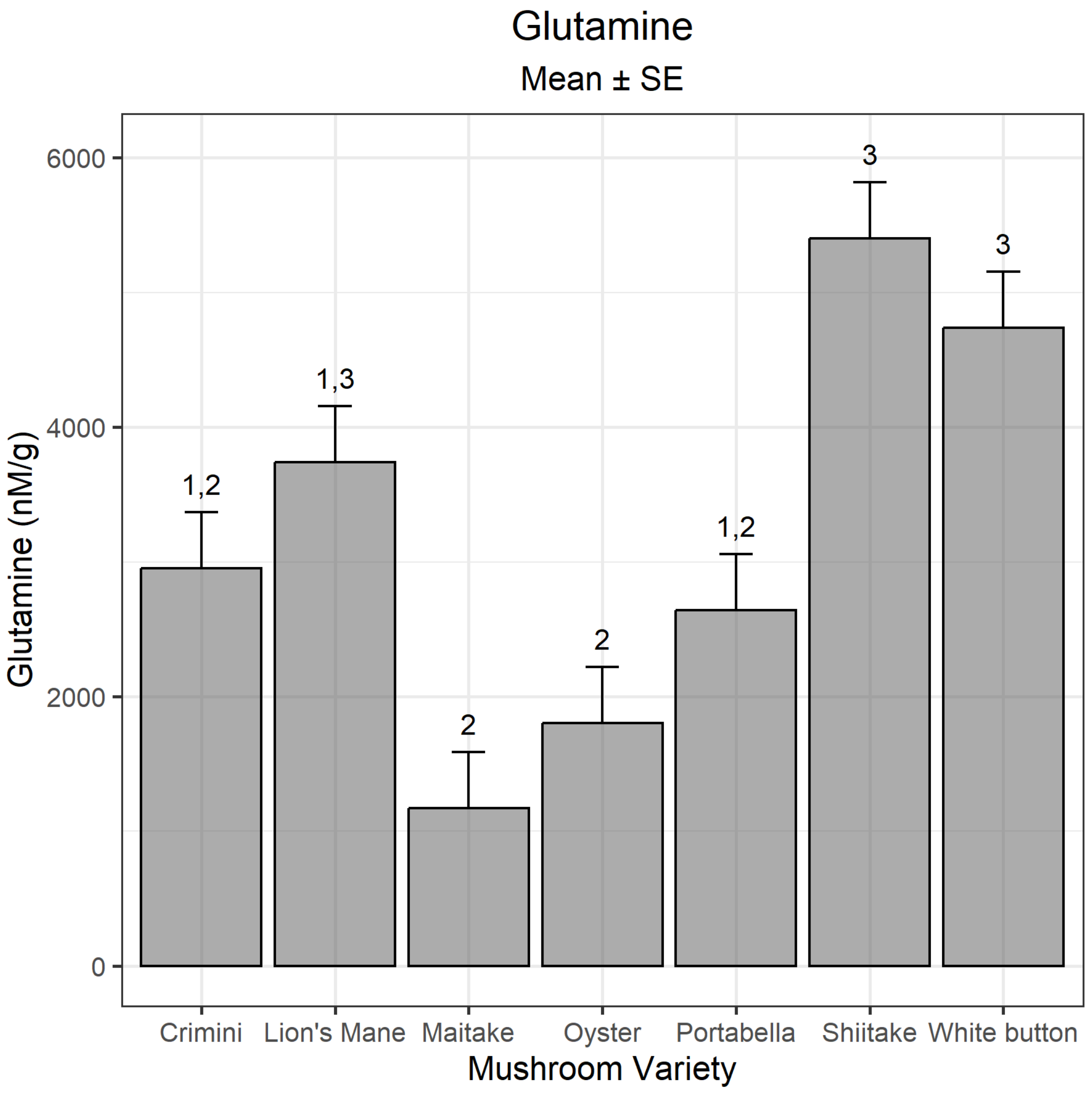

Data are pooled mean ± SE. Different numbers denote significance ($p < 0.05$).

Glycine

Mushroom Variety	Emmean	SE	df	Lower.CL	Upper.CL	Groups
Crimini	616.28	70.83	35	472.48	760.08	2
Lion's Mane	136.20	70.83	35	-7.60	280.00	1
Maitake	74.82	70.83	35	-68.98	218.62	1
Oyster	120.78	70.83	35	-23.02	264.58	1
Portabella	754.54	70.83	35	610.74	898.34	2
Shiitake	358.46	70.83	35	214.66	502.26	1
White button	870.95	70.83	35	727.14	1014.75	2

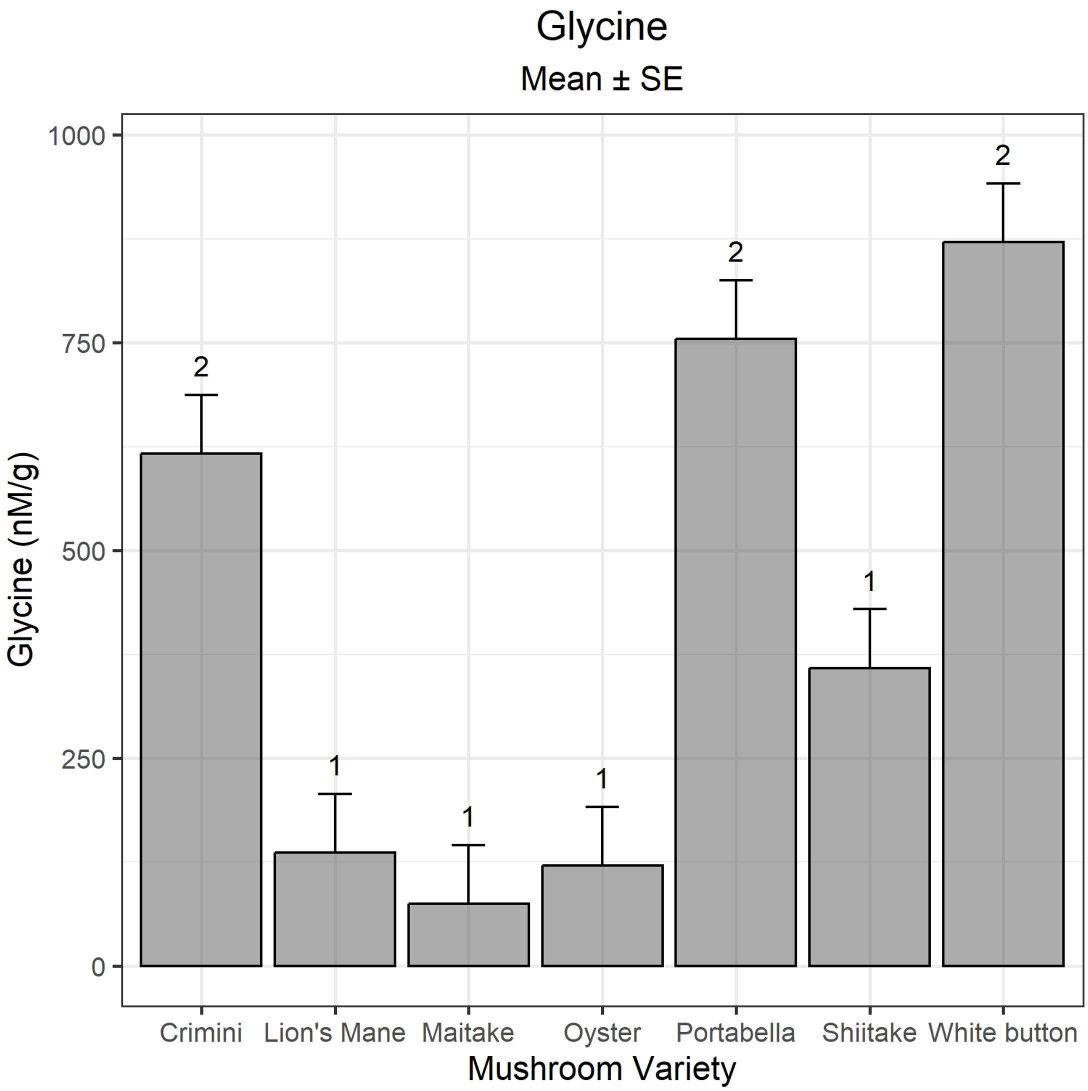

Data are pooled mean ± SE. Different numbers denote significance (p <0.05).

Serine

Mushroom Variety	Emmean	SE	df	Lower.CL	Upper.CL	Groups
Crimini	941.97	100.17	35	738.61	1145.33	2
Lion's Mane	385.91	100.17	35	182.55	589.27	1
Maitake	362.48	100.17	35	159.12	565.84	1
Oyster	367.36	100.17	35	164.00	570.72	1
Portabella	1082.93	100.17	35	879.57	1286.29	2,3
Shiitake	704.52	100.17	35	501.16	907.88	1,2
White button	1398.44	100.17	35	1195.08	1601.80	3

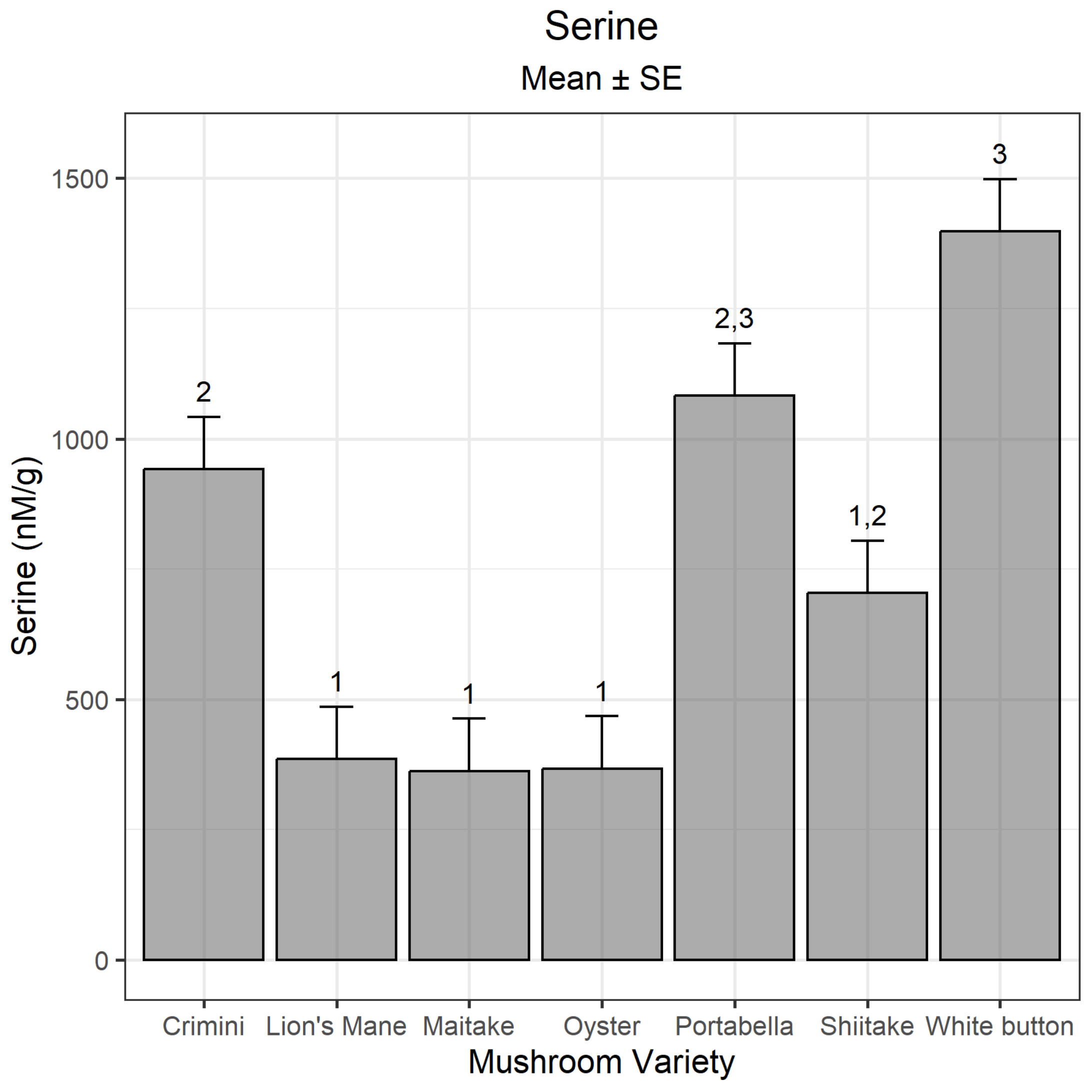

Data are pooled mean ± SE. Different numbers denote significance ($p < 0.05$).

Threonine

Mushroom Variety	Emmean	SE	df	Lower.CL	Upper.CL	Groups
Crimini	841.82	77.70	35	684.08	999.57	2,3
Lion's Mane	254.82	77.70	35	97.07	412.56	1
Maitake	295.96	77.70	35	138.22	453.70	1
Oyster	292.88	77.70	35	135.14	450.62	1
Portabella	798.98	77.70	35	641.23	956.72	2
Shiitake	670.45	77.70	35	512.71	828.20	2
White button	1165.02	77.70	35	1007.28	1322.77	3

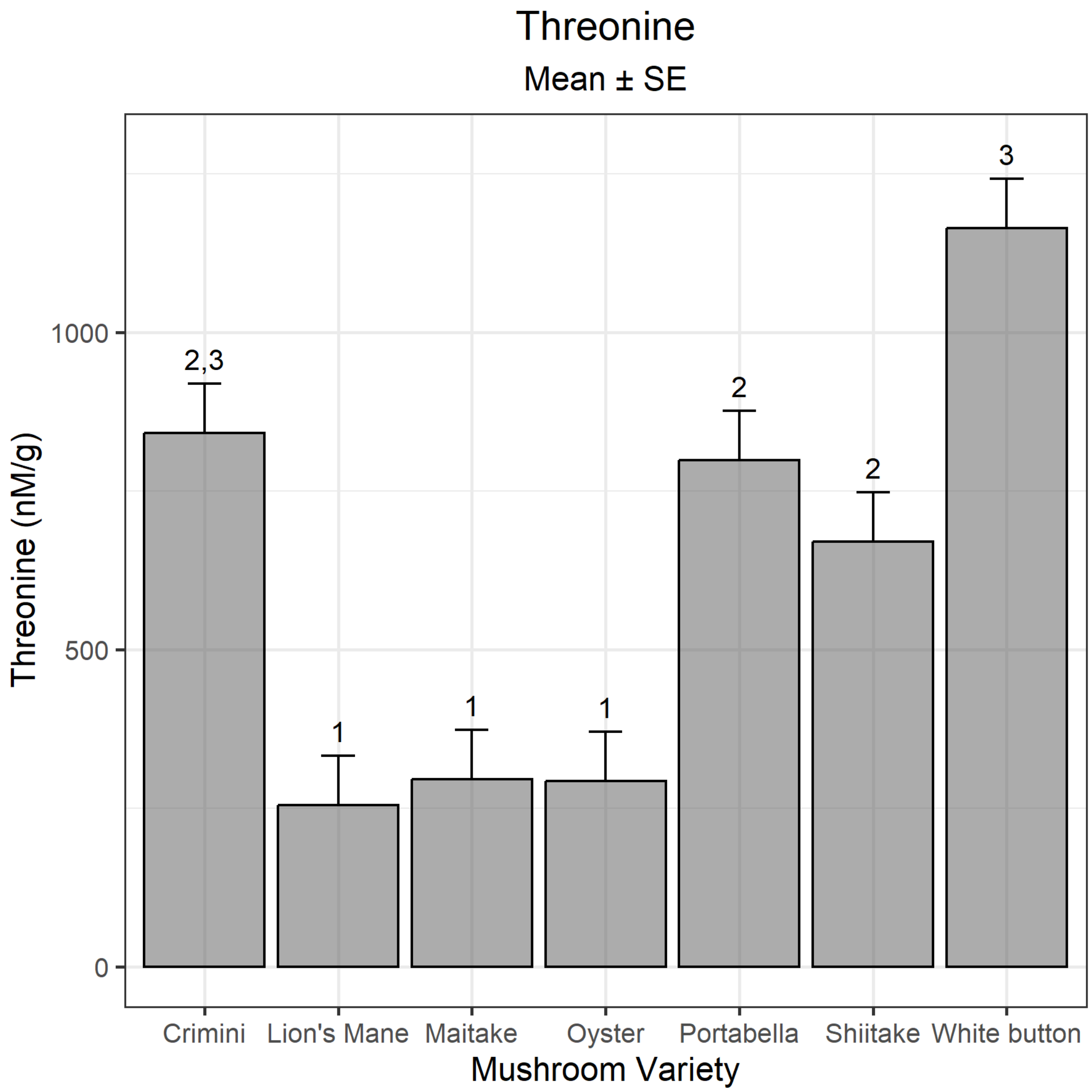

Data are pooled mean ± SE. Different numbers denote significance ($p < 0.05$).

Tyrosine

Mushroom Variety	Emmean	SE	df	Lower.CL	Upper.CL	Groups
Crimini	510.52	64.82	33	378.64	642.40	3
Lion's Mane	208.12	65.60	33	74.66	341.59	1,2
Maitake	211.73	65.60	33	78.27	345.20	1,2
Oyster	429.86	65.60	33	296.39	563.32	1,3
Portabella	346.45	64.82	33	214.58	478.33	1,2,3
Shiitake	138.92	65.60	33	5.45	272.38	2
White button	360.39	64.82	33	228.51	492.27	1,2,3

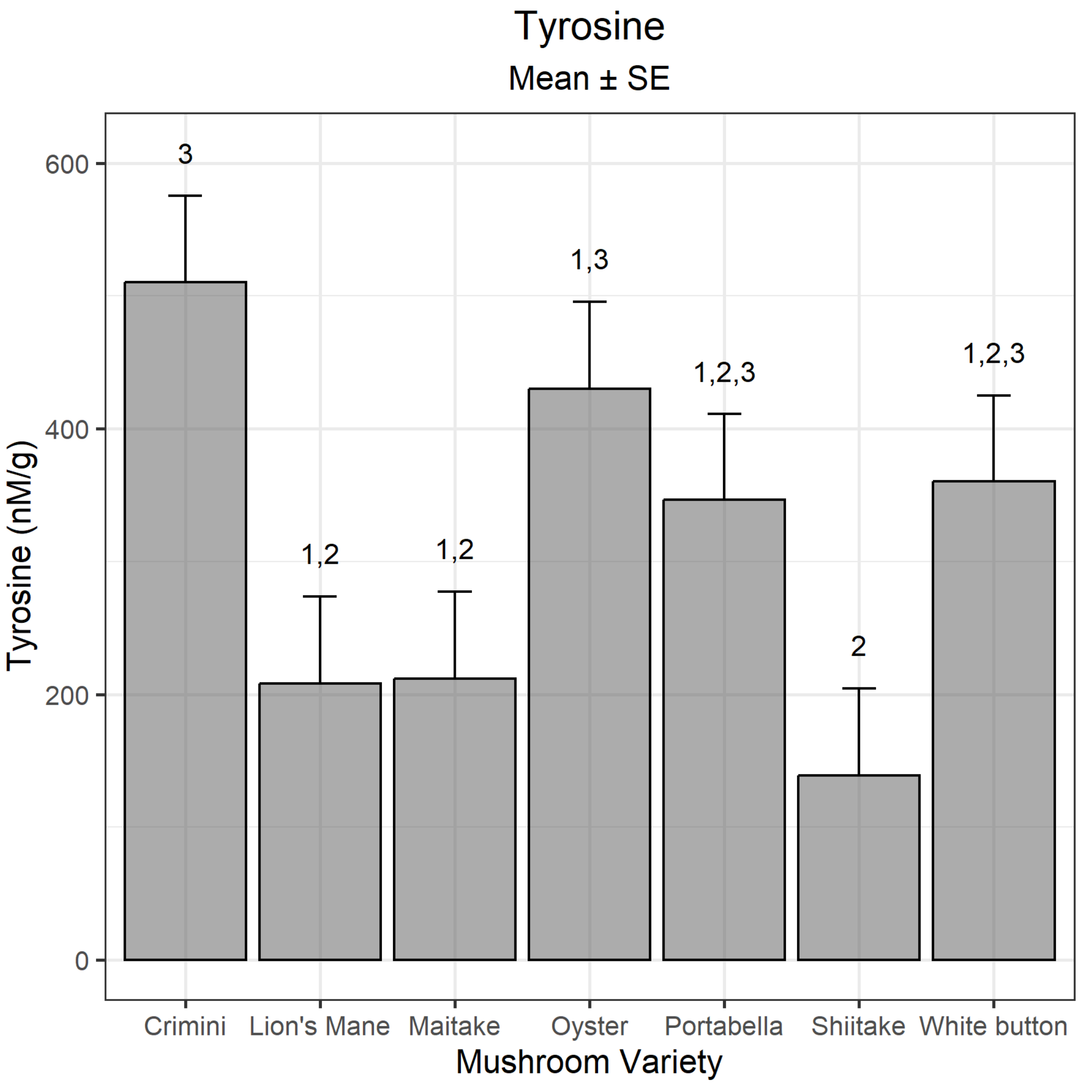

Data are pooled mean ± SE. Different numbers denote significance ($p < 0.05$).

1-methylhistidine

Mushroom Variety	Emmean	SE	df	Lower.CL	Upper.CL	Groups
Crimini	20.68	2.34	35	15.93	25.42	1
Lion's Mane	19.11	2.34	35	14.37	23.85	1
Maitake	16.36	2.34	35	11.62	21.10	1
Oyster	16.22	2.34	35	11.48	20.97	1
Portabella	24.72	2.34	35	19.98	29.47	1
Shiitake	17.24	2.34	35	12.49	21.98	1
White button	26.41	2.34	35	21.66	31.15	1

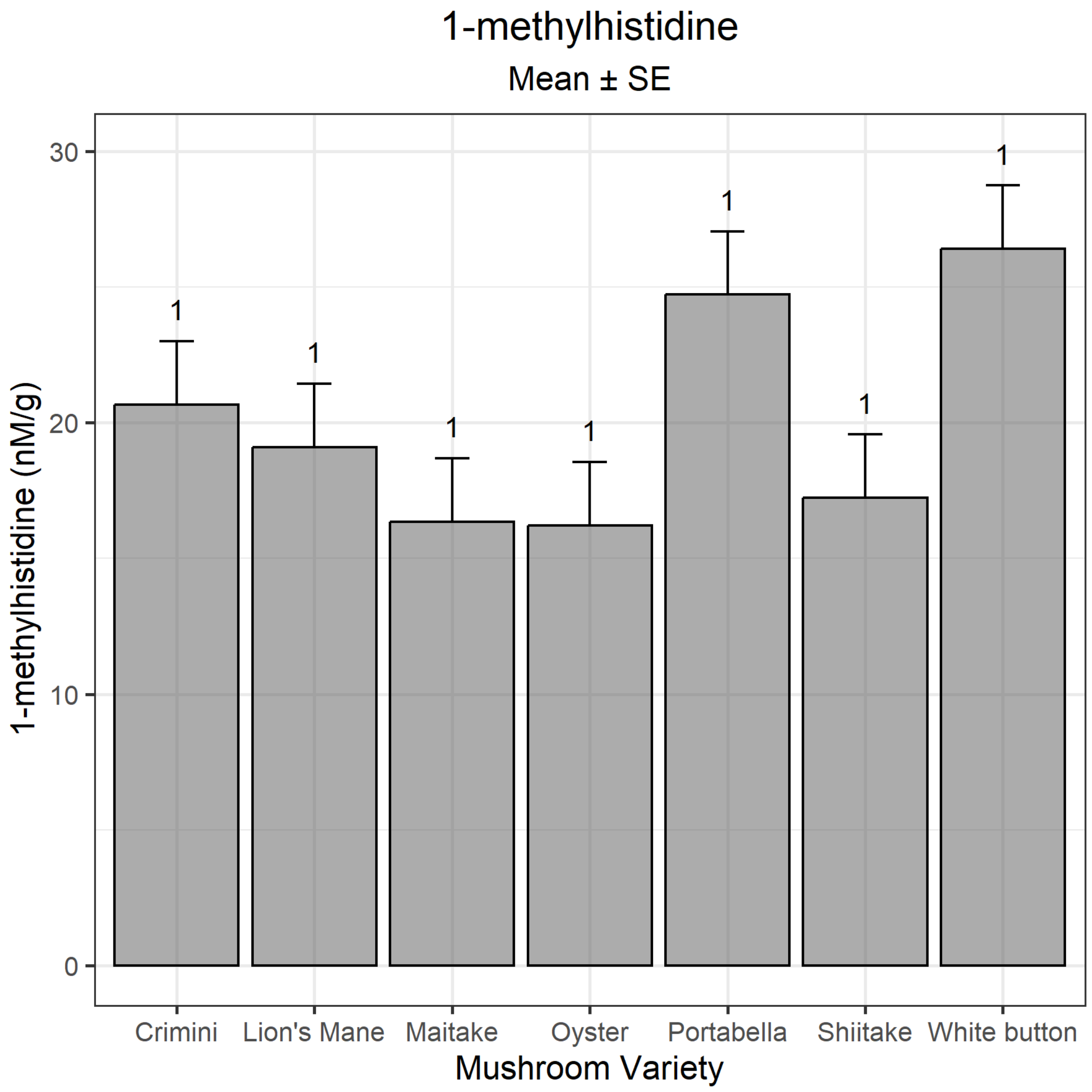

Data are pooled mean ± SE. Different numbers denote significance ($p < 0.05$).

Cystathionine

Mushroom Variety	Emmean	SE	df	Lower.CL	Upper.CL	Groups
Crimini	151.29	16.18	28	118.16	184.43	2,3
Lion's Mane	63.84	16.18	28	30.70	96.97	1
Maitake	0.00	16.18	28	-33.14	33.14	1
Oyster	160.74	16.18	28	127.61	193.88	2
Portabella	84.41	16.18	28	51.27	117.54	3
Shiitake	143.41	16.18	28	110.27	176.54	2,3
White button	180.82	16.18	28	147.68	213.95	2

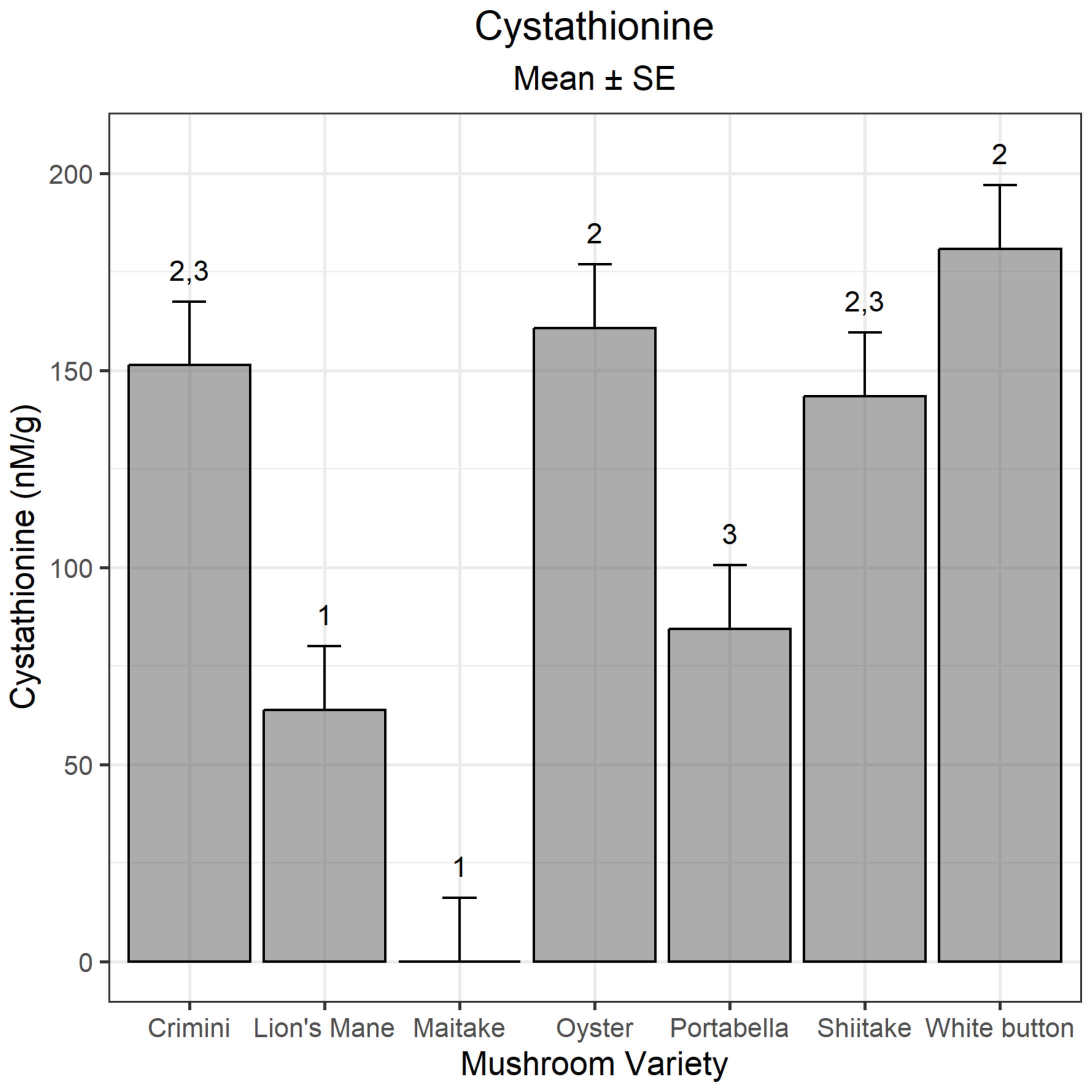

Data are pooled mean ± SE. Different numbers denote significance ($p < 0.05$).

Ergothioneine

Mushroom Variety	Emmean	SE	df	Lower.CL	Upper.CL	Groups
Crimini	141.85	33.79	28	72.63	211.07	2
Lion's Mane	804.69	33.79	28	735.47	873.90	1
Maitake	121.45	33.79	28	52.23	190.67	2
Oyster	456.11	33.79	28	386.89	525.33	3
Portabella	115.22	33.79	28	46.00	184.44	2
Shiitake	226.65	33.79	28	157.43	295.87	2
White button	166.04	33.79	28	96.82	235.26	2

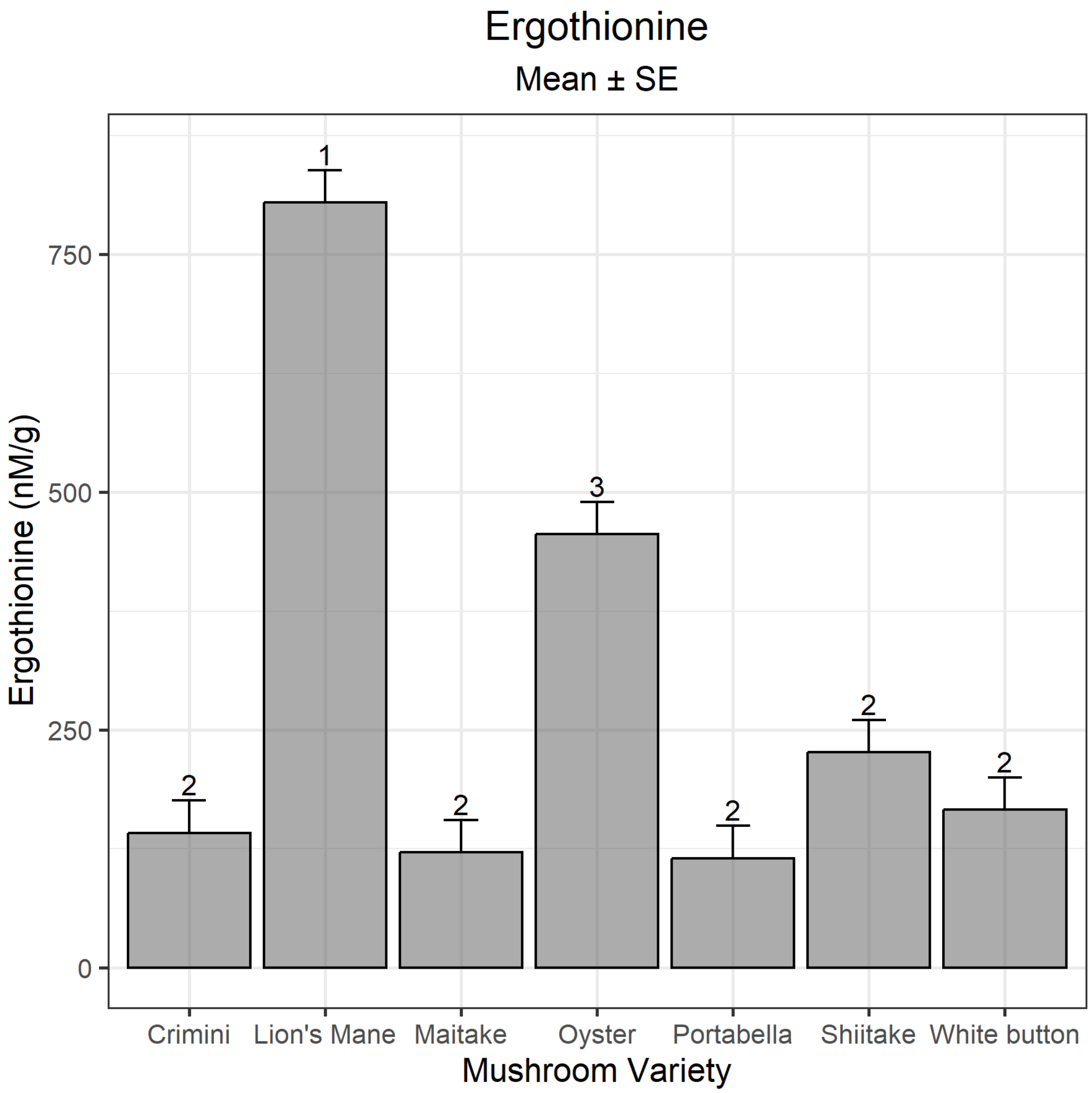

Data are pooled mean ± SE. Different numbers denote significance ($p < 0.05$).

g-aminobutyric acid

Mushroom Variety	Emmean	SE	df	Lower.CL	Upper.CL	Groups
Crimini	84.55	31.08	35	21.46	147.64	1,2
Lion's Mane	181.78	31.08	35	118.70	244.87	1,3,4
Maitake	30.13	31.08	35	-32.96	93.22	2
Oyster	53.33	31.08	35	-9.75	116.42	1,2
Portabella	232.02	31.08	35	168.93	295.11	3,4
Shiitake	98.81	31.08	35	35.72	161.90	1,2,3
White button	256.98	31.08	35	193.89	320.07	4

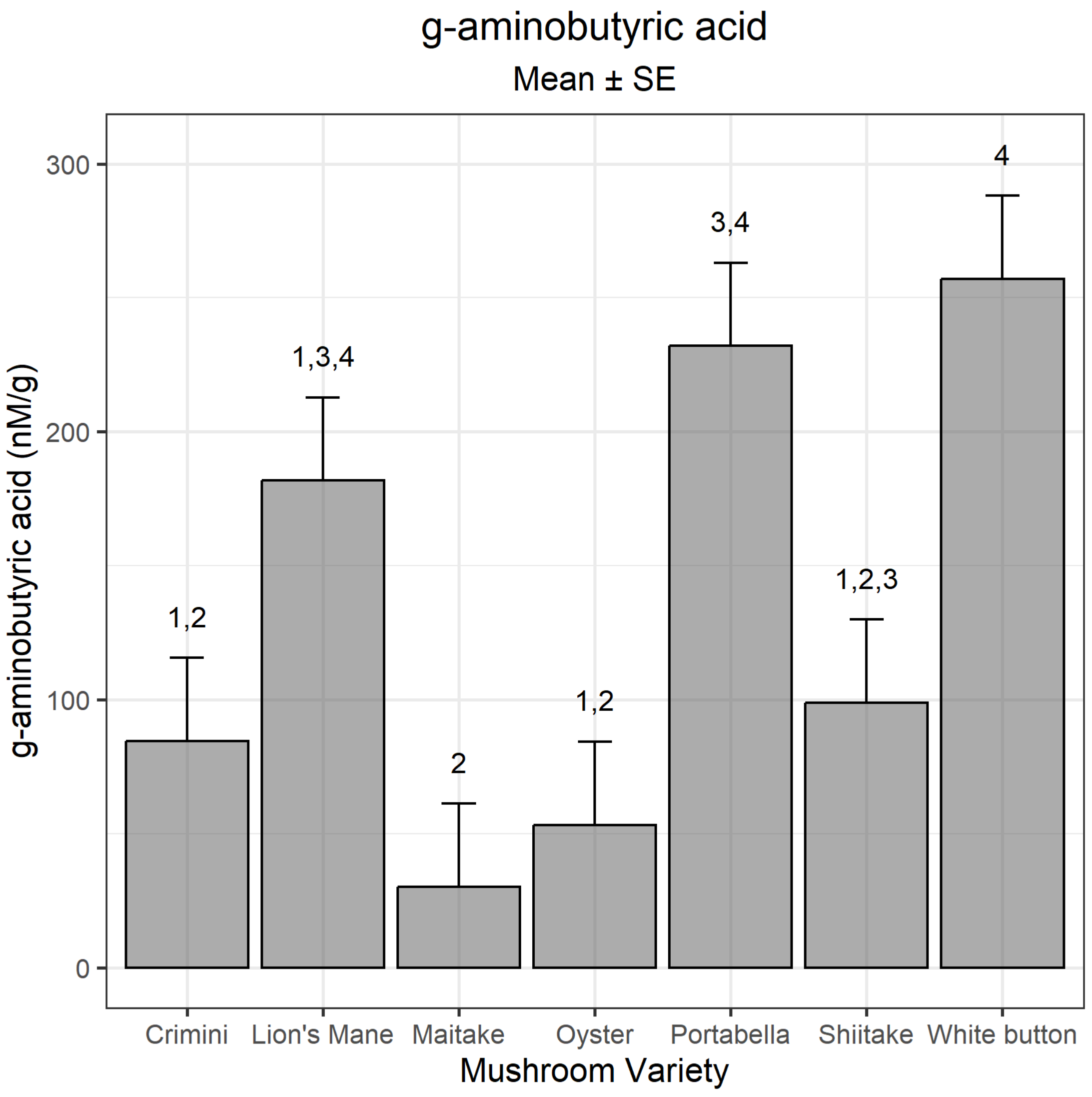

Data are pooled mean ± SE. Different numbers denote significance ($p < 0.05$).

Hydroxyproline

Mushroom Variety	Emmean	SE	df	Lower.CL	Upper.CL	Groups
Crimini	28.84	5.07	33	18.53	39.15	2
Lion's Mane	12.70	5.13	33	2.27	23.14	1
Maitake	9.42	5.13	33	-1.02	19.85	1
Oyster	0.98	5.13	33	-9.45	11.42	1
Portabella	41.73	5.07	33	31.42	52.04	2
Shiitake	-5.66	5.13	33	-16.10	4.77	1
White button	45.80	5.07	33	35.49	56.12	2

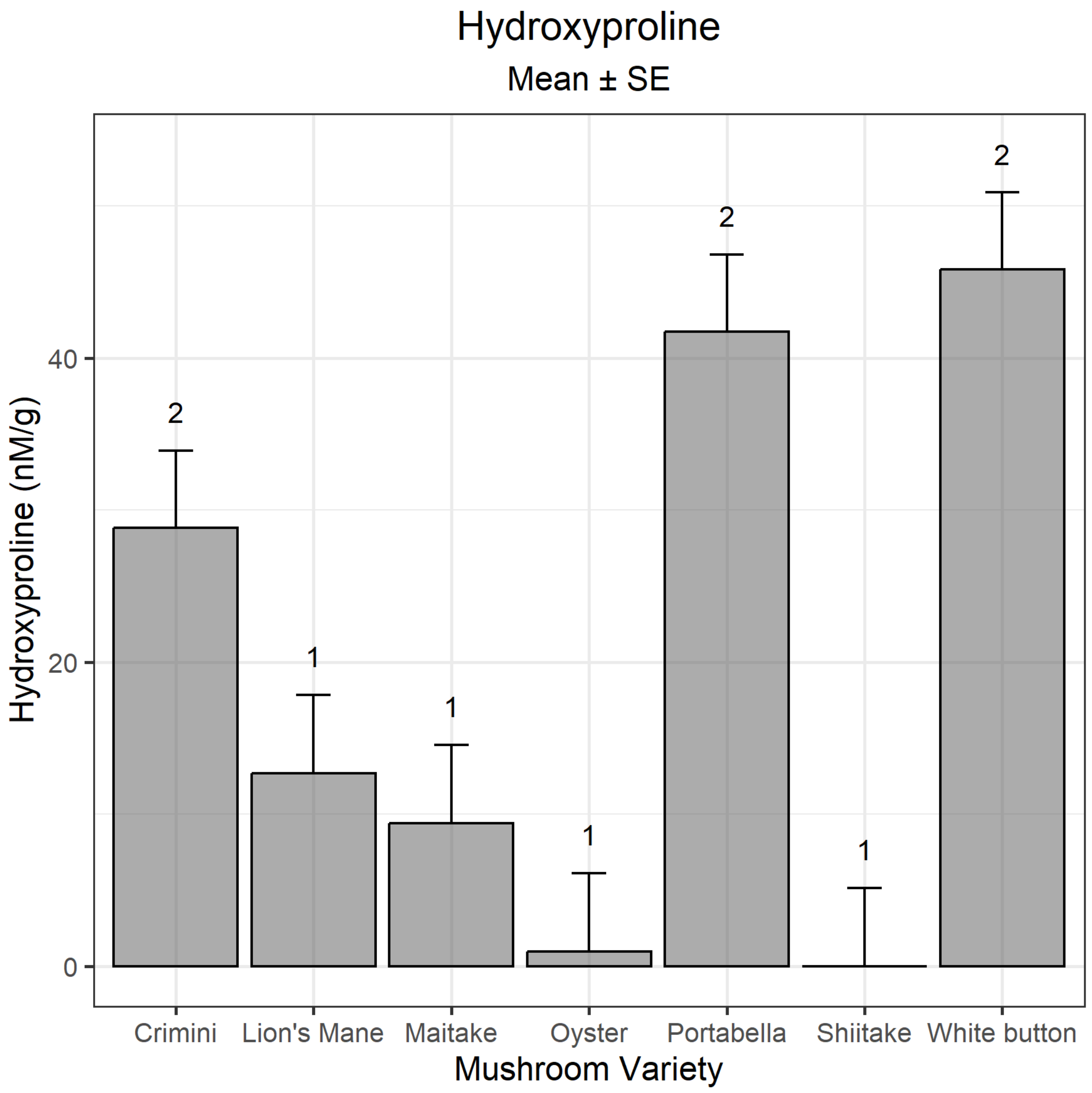

Data are pooled mean ± SE. Different numbers denote significance ($p < 0.05$).

L-aminoadipic acid

Mushroom Variety	Emmean	SE	df	Lower.CL	Upper.CL	Groups
Crimini	207.07	20.91	28	164.23	249.91	3
Lion's Mane	57.16	20.91	28	14.32	100.00	1
Maitake	149.58	20.91	28	106.74	192.42	1,3
Oyster	421.99	20.91	28	379.15	464.83	2
Portabella	176.48	20.91	28	133.64	219.32	3
Shiitake	67.51	20.91	28	24.67	110.35	1
White button	162.61	20.91	28	119.76	205.45	3

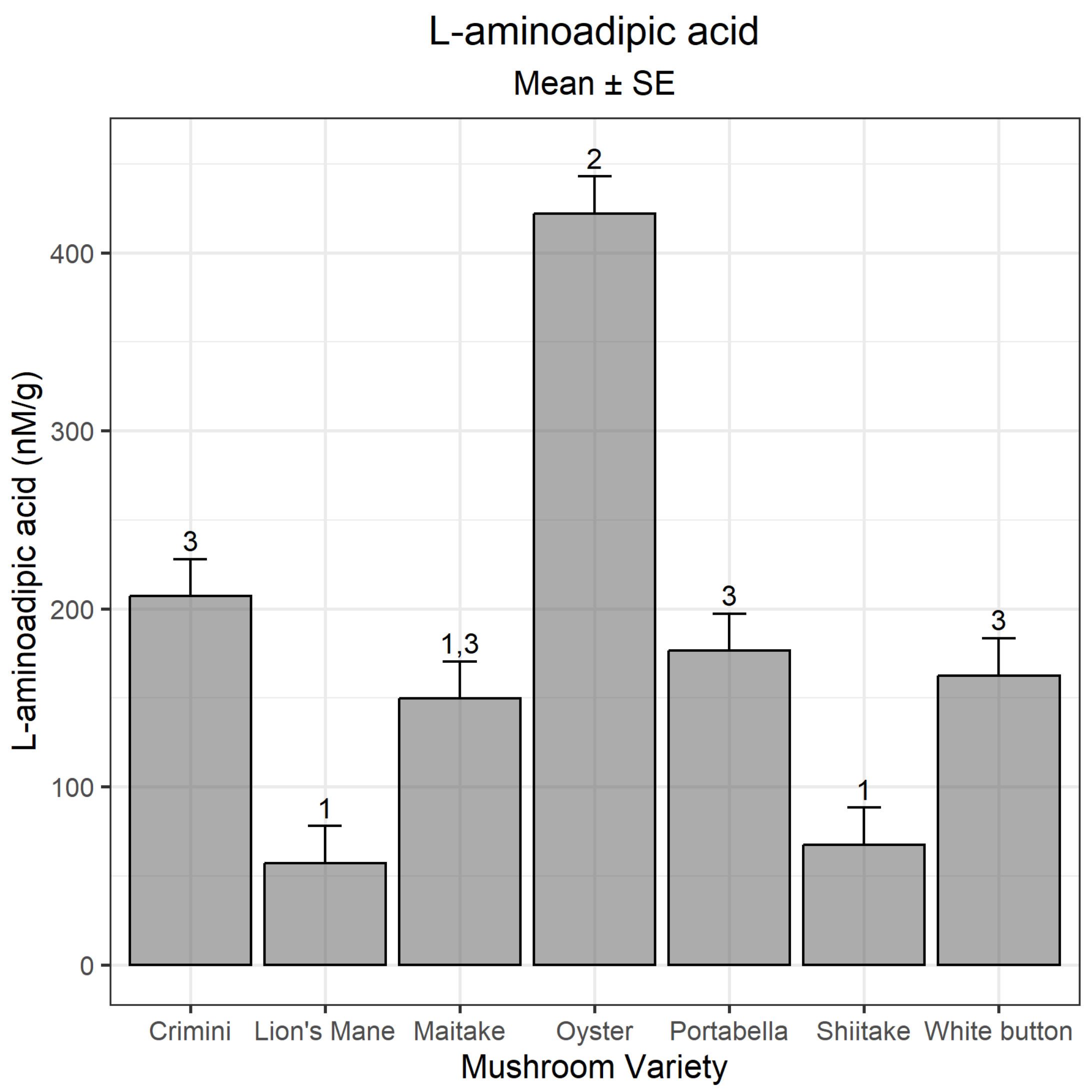

Data are pooled mean ± SE. Different numbers denote significance ($p < 0.05$).

Ornithine

Mushroom Variety	Emmean	SE	df	Lower.CL	Upper.CL	Groups
Crimini	1151.85	140.71	28	863.62	1440.08	2
Lion's Mane	61.31	140.71	28	-226.92	349.53	1
Maitake	1643.44	140.71	28	1355.21	1931.67	2,3
Oyster	358.38	140.71	28	70.15	646.61	1
Portabella	1170.97	140.71	28	882.74	1459.19	2
Shiitake	1860.73	140.71	28	1572.50	2148.96	3
White button	1095.21	140.71	28	806.98	1383.44	2

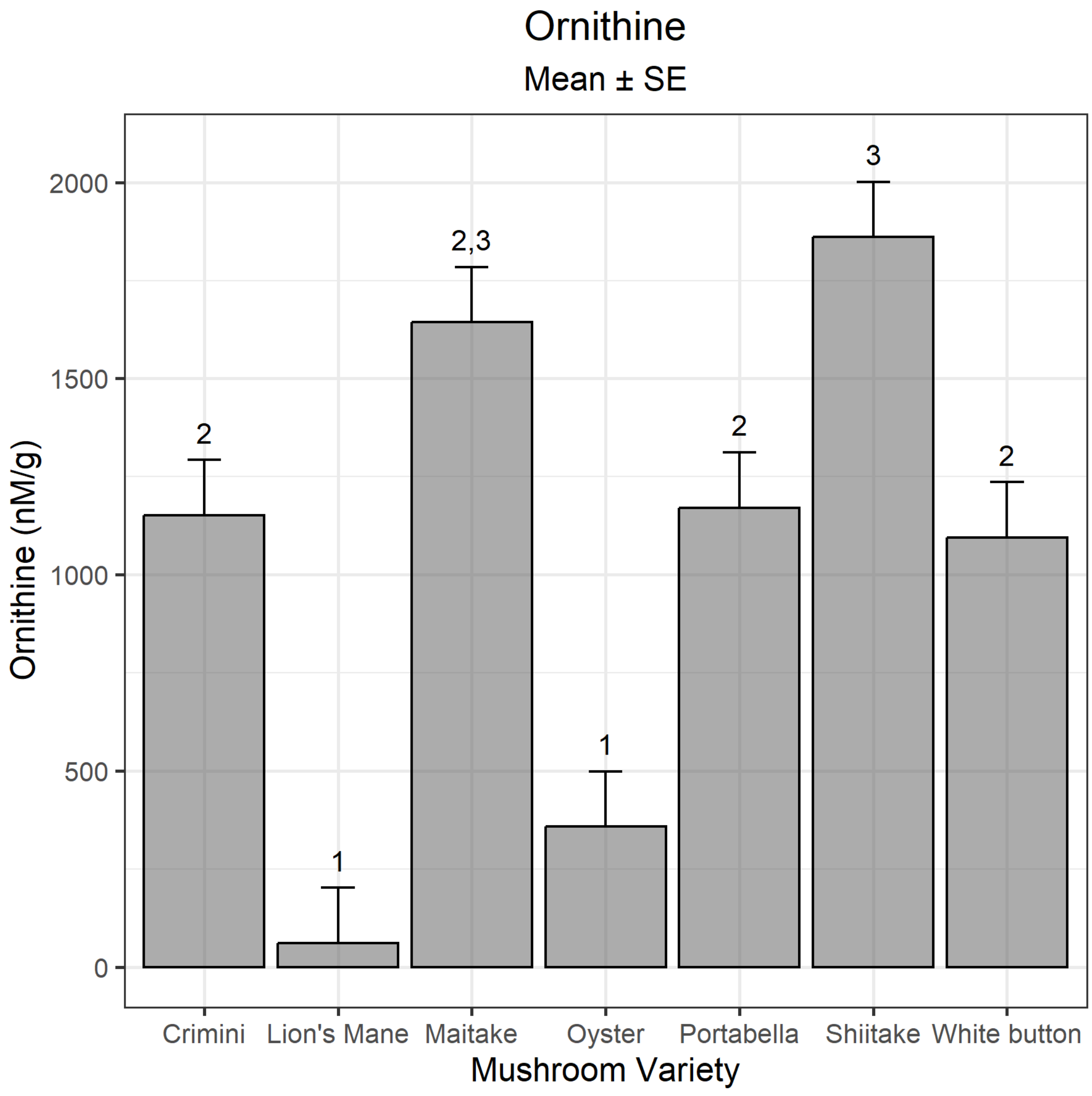

Data are pooled mean ± SE. Different numbers denote significance ($p < 0.05$).

Oxidized glutathione

Mushroom Variety	Emmean	SE	df	Lower.CL	Upper.CL	Groups
Crimini	10.05	4.21	35	1.50	18.60	2
Lion's Mane	20.41	4.21	35	11.85	28.96	1,2
Maitake	32.43	4.21	35	23.88	40.98	1
Oyster	7.01	4.21	35	-1.54	15.56	2
Portabella	10.35	4.21	35	1.80	18.91	2
Shiitake	7.28	4.21	35	-1.27	15.83	2
White button	9.00	4.21	35	0.45	17.55	2

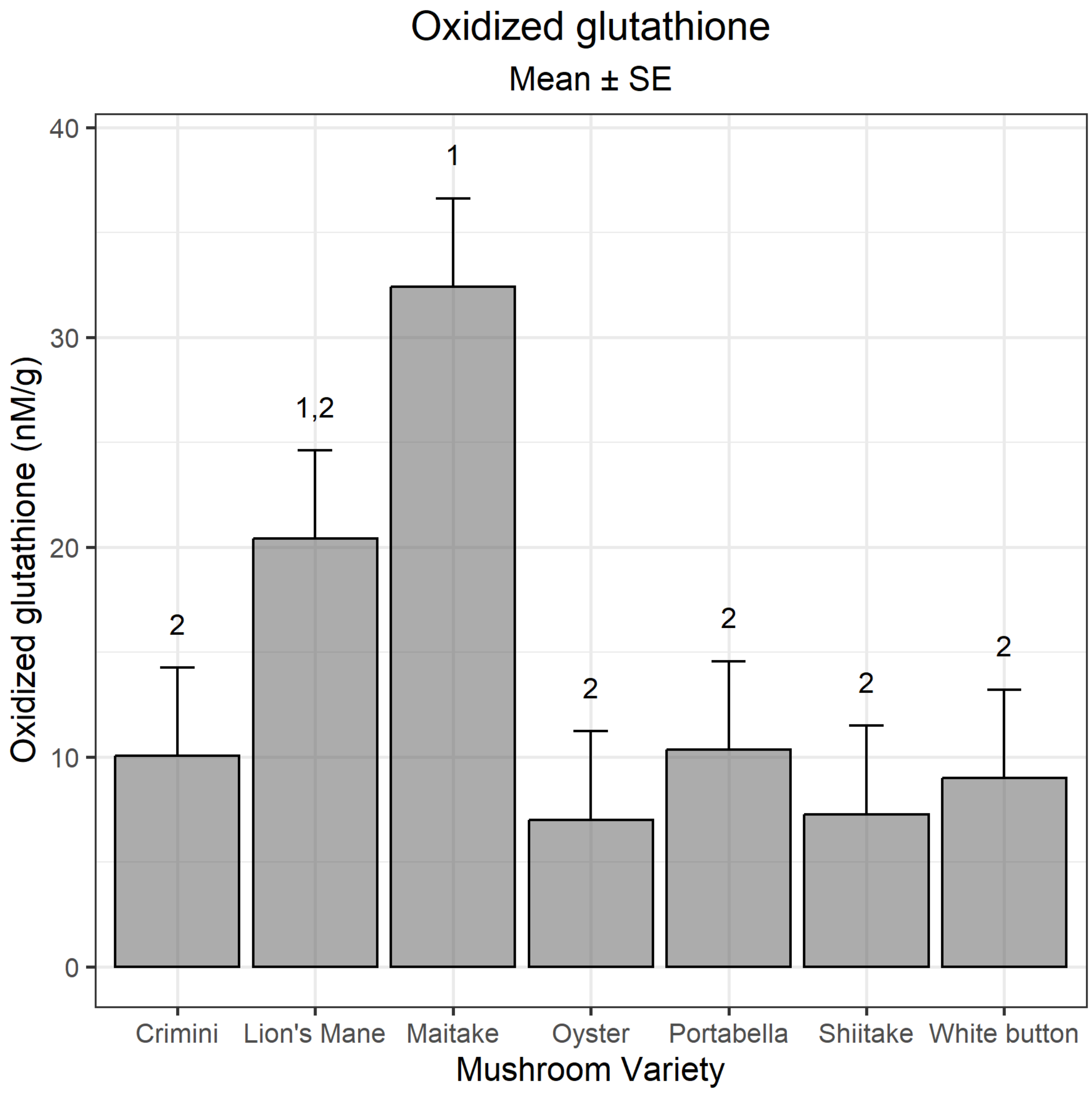

Data are pooled mean ± SE. Different numbers denote significance ($p < 0.05$).

Reduced glutathione

Mushroom Variety	Emmean	SE	df	Lower.CL	Upper.CL	Groups
Crimini	673.84	541.29	35	-425.04	1772.71	1
Lion's Mane	217.49	541.29	35	-881.39	1316.36	1
Maitake	3738.46	541.29	35	2639.59	4837.34	2
Oyster	3844.43	541.29	35	2745.56	4943.31	2
Portabella	516.32	541.29	35	-582.56	1615.19	1
Shiitake	606.99	541.29	35	-491.89	1705.86	1
White button	1491.99	541.29	35	393.11	2590.86	1,2

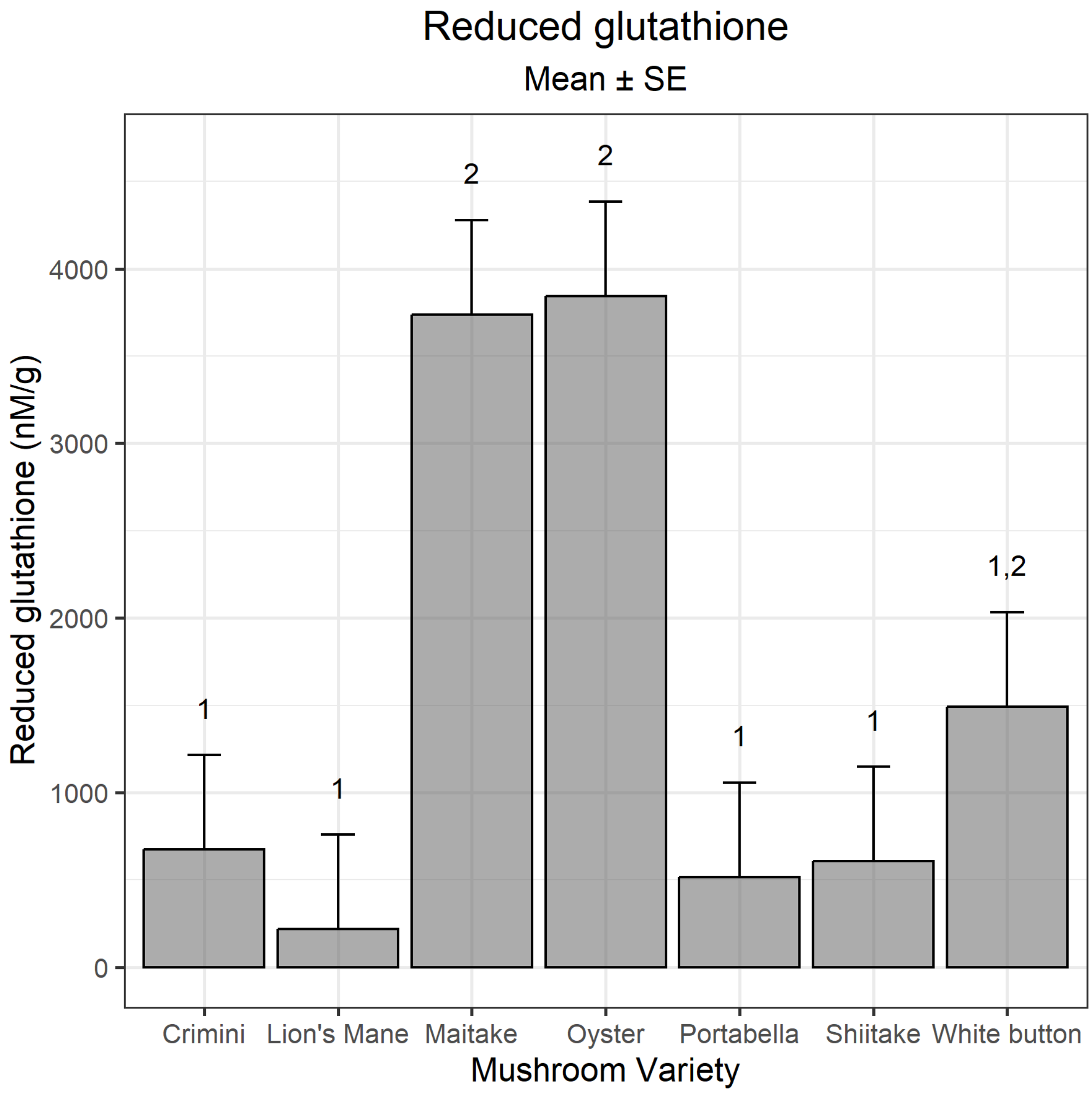

Data are pooled mean $\pm$ SE. Different numbers denote significance ($p < 0.05$).

Sarcosine

Mushroom Variety	Emmean	SE	df	Lower.CL	Upper.CL	Groups
Crimini	56.63	3.55	28	49.35	63.91	3,4
Lion's Mane	20.30	3.55	28	13.03	27.58	1
Maitake	0.00	3.55	28	-7.28	7.28	2
Oyster	59.53	3.55	28	52.26	66.81	3,4
Portabella	68.70	3.55	28	61.42	75.98	3
Shiitake	44.29	3.55	28	37.01	51.56	4
White button	57.69	3.55	28	50.42	64.97	3,4

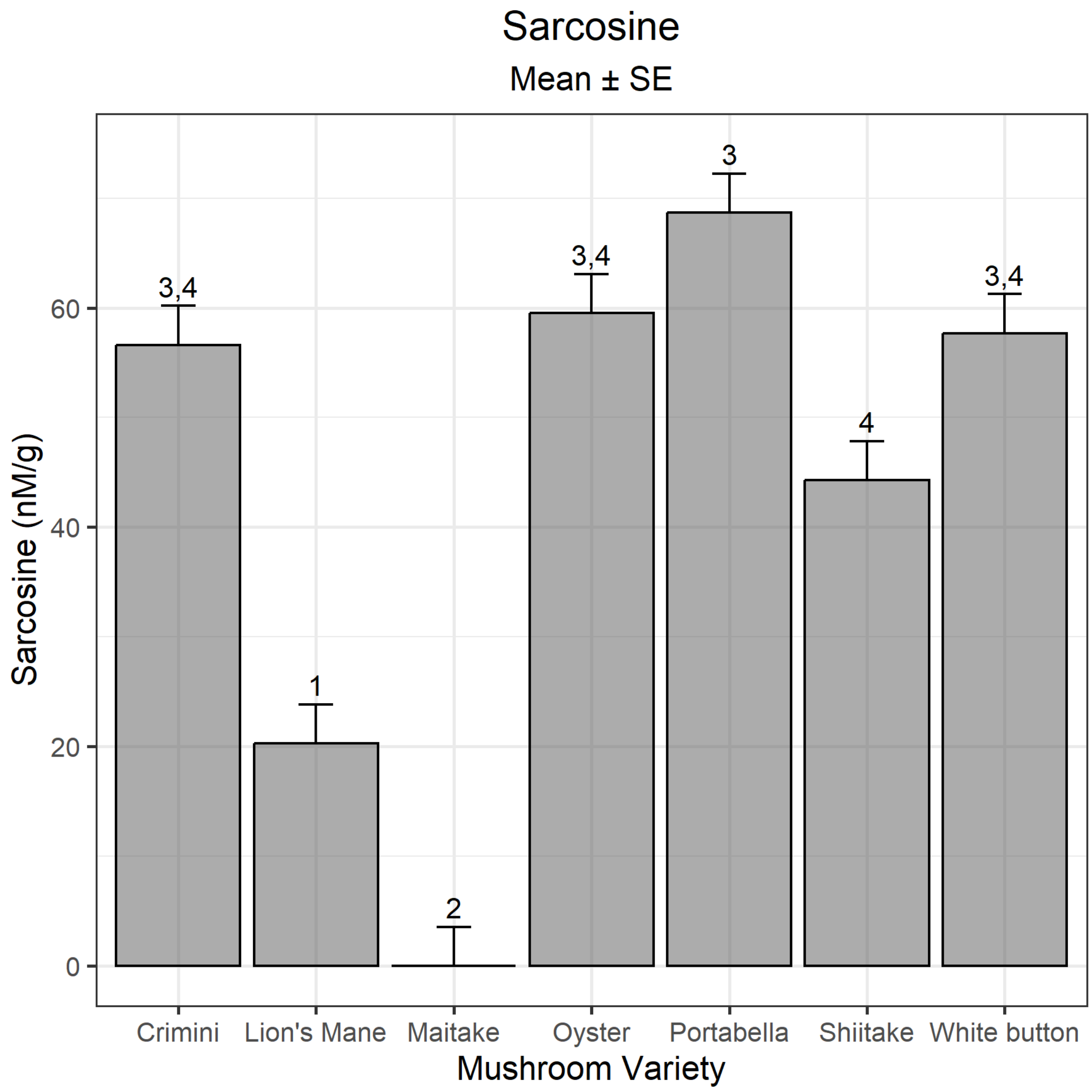

Data are pooled mean ± SE. Different numbers denote significance ($p < 0.05$).
