# Supplementary material for: Metabolomics Profiling of White Button, Crimini, Portabella, Lion’s Mane, Maitake, Oyster, and Shiitake Mushrooms Using Untargeted Metabolomics and Targeted Amino Acid Analysis
Source: Foods. 2023 Aug 8;12(16):2985. doi: 10.3390/foods12162985 (PMC10453450; doi:10.3390/foods12162985)
Supplement: Supplementary file 1 [file foods-12-02985-s001.zip › File S5. Figures S1, S2, S3 .pdf]

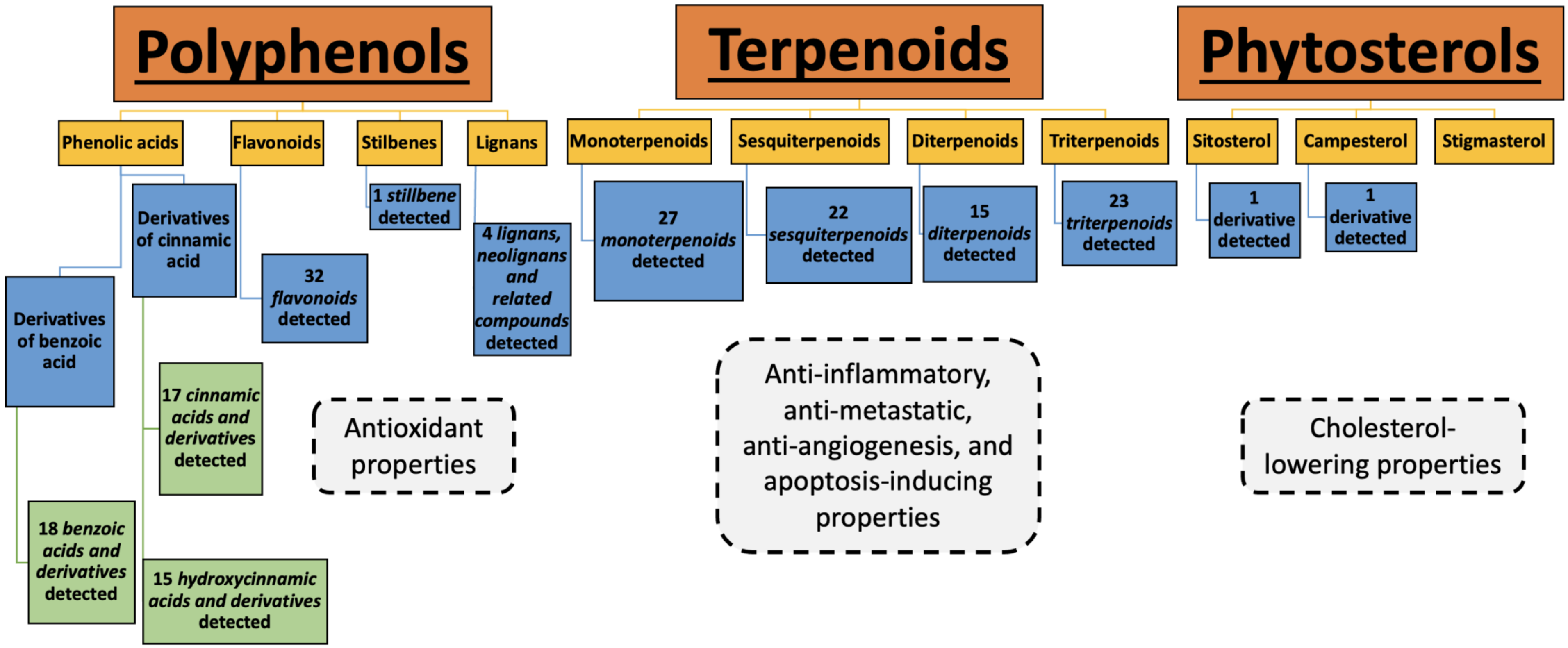

Figure S1. Select bioactive compounds detected in sample replicates of seven mushroom varieties.

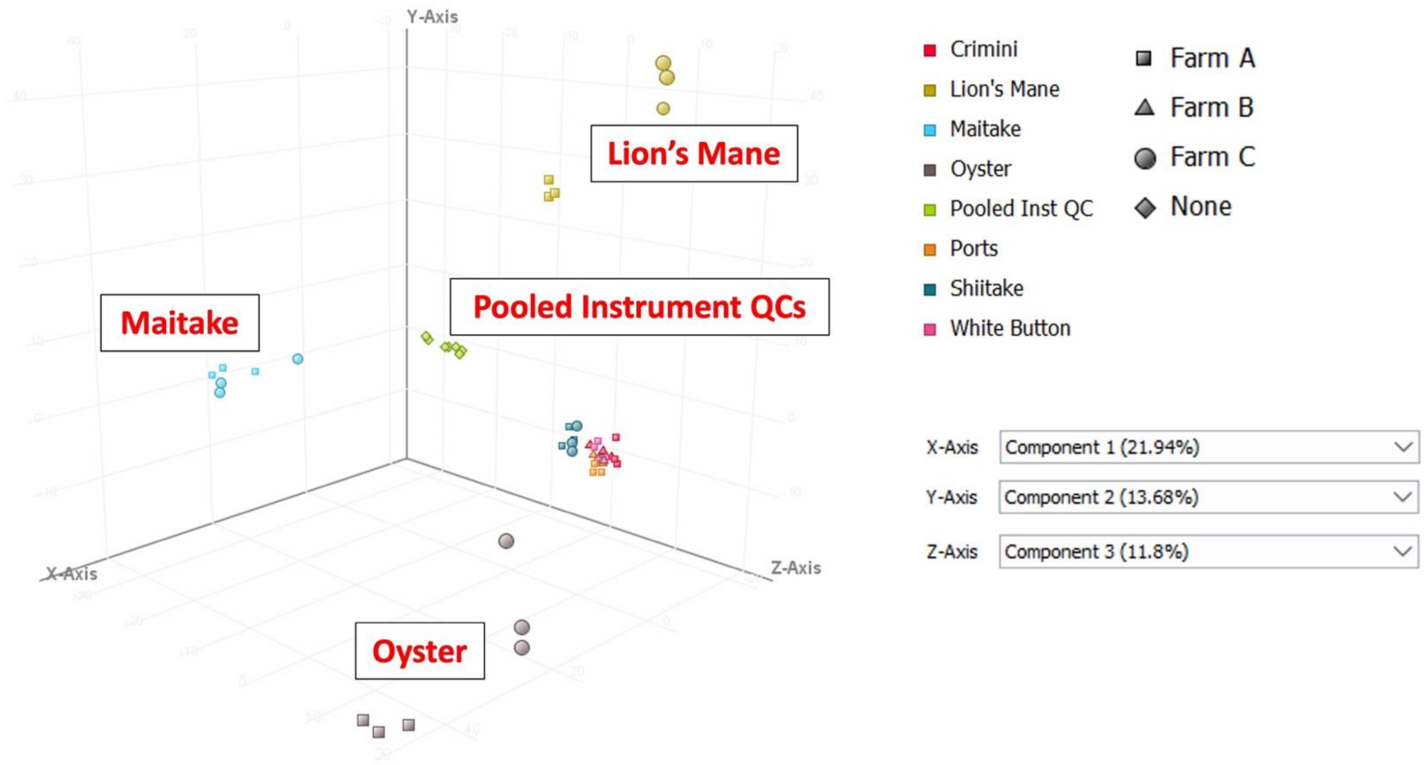

Figure S2. Principal Component Analysis (PCA) using data from the hydrophobic fraction of seven mushroom varieties along with the pooled instrument QCs. Component 1, which explains 21.94% of the variation, is shown on the x-axis; component 2, which explains 13.68% of the variation, is shown on the y-axis; and component 3, which explains 11.8% of the variation, is shown on the z-axis.

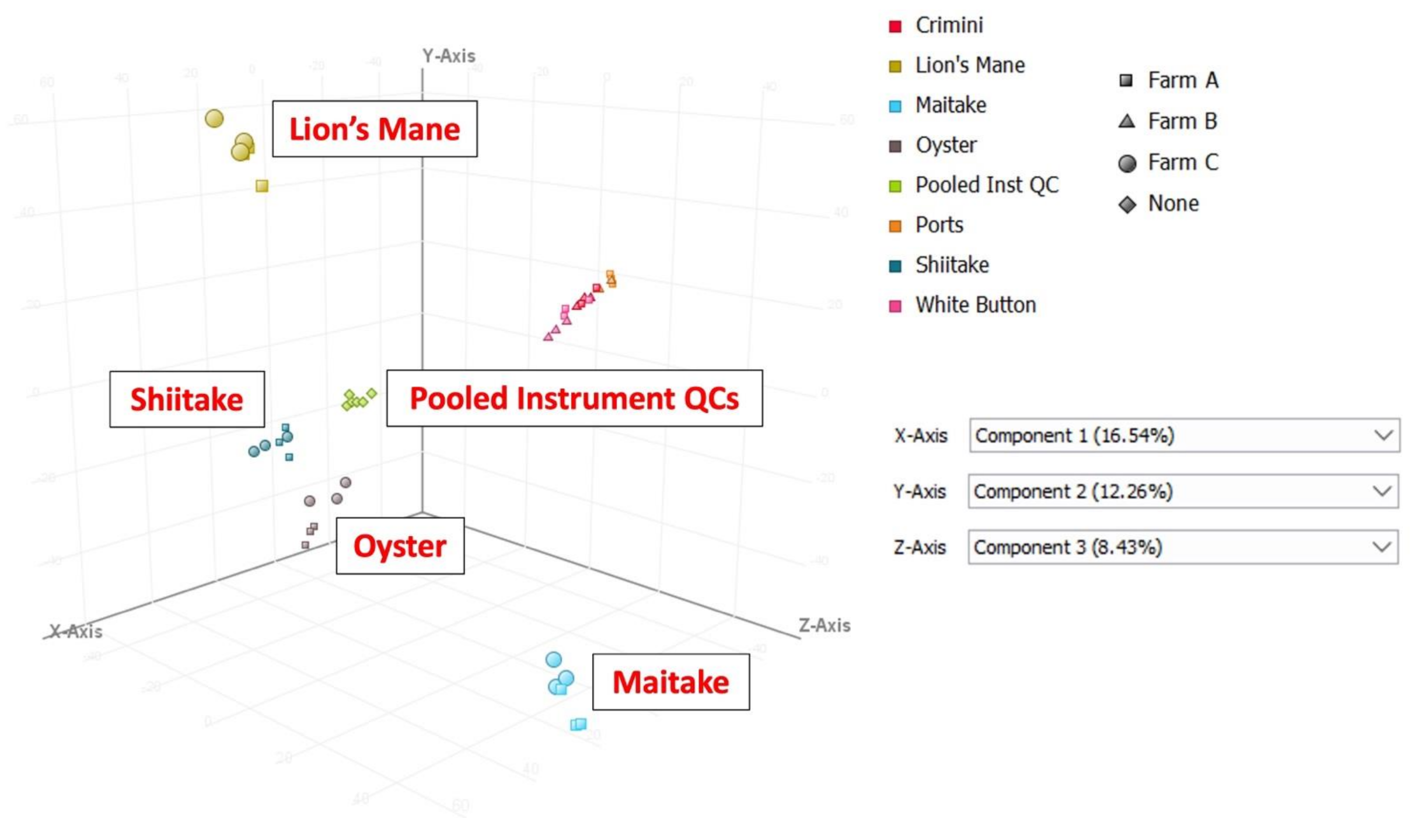

Figure S3. Principal Component Analysis (PCA) using data from the hydrophilic fraction of seven mushroom varieties along with the pooled instrument QCs. Component 1, which explains 16.54% of the variation, is shown on the x-axis; component 2, which explains 12.26% of the variation, is shown on the y-axis; and component 3, which explains 8.43% of the variation, is shown on the z-axis.
